# RNA-seq Analysis Reveals Unique Transcriptome Signatures in Systemic Lupus Erythematosus Patients with Distinct Autoantibody Specificities

**DOI:** 10.1371/journal.pone.0166312

**Published:** 2016-11-11

**Authors:** Richa Rai, Sudhir Kumar Chauhan, Vikas Vikram Singh, Madhukar Rai, Geeta Rai

**Affiliations:** 1 Department of Molecular and Human Genetics, Institute of Science, Banaras Hindu University, Varanasi, Uttar Pradesh, India; 2 Department of Medicine, Institute of Medical Sciences, Banaras Hindu University, Varanasi, Uttar Pradesh, India; Instituto Nacional de Ciencias Medicas y Nutricion Salvador Zubiran, MEXICO

## Abstract

Systemic lupus erythematosus (SLE) patients exhibit immense heterogeneity which is challenging from the diagnostic perspective. Emerging high throughput sequencing technologies have been proved to be a useful platform to understand the complex and dynamic disease processes. SLE patients categorised based on autoantibody specificities are reported to have differential immuno-regulatory mechanisms. Therefore, we performed RNA-seq analysis to identify transcriptomics of SLE patients with distinguished autoantibody specificities. The SLE patients were segregated into three subsets based on the type of autoantibodies present in their sera (anti-dsDNA^+^ group with anti-dsDNA autoantibody alone; anti-ENA^+^ group having autoantibodies against extractable nuclear antigens (ENA) only, and anti-dsDNA^+^ENA^+^ group having autoantibodies to both dsDNA and ENA). Global transcriptome profiling for each SLE patients subsets was performed using Illumina® Hiseq-2000 platform. The biological relevance of dysregulated transcripts in each SLE subsets was assessed by ingenuity pathway analysis (IPA) software. We observed that dysregulation in the transcriptome expression pattern was clearly distinct in each SLE patients subsets. IPA analysis of transcripts uniquely expressed in different SLE groups revealed specific biological pathways to be affected in each SLE subsets. Multiple *cytokine signaling* pathways were specifically dysregulated in anti-dsDNA^+^ patients whereas *Interferon signaling* was predominantly dysregulated in anti-ENA^+^ patients. In anti-dsDNA^+^ENA^+^ patients *regulation of actin based motility by Rho* pathway was significantly affected. The granulocyte gene signature was a common feature to all SLE subsets; however, anti-dsDNA^+^ group showed relatively predominant expression of these genes. Dysregulation of Plasma cell related transcripts were higher in anti-dsDNA^+^ and anti-ENA^+^ patients as compared to anti-dsDNA^+^ ENA^+^. Association of specific canonical pathways with the uniquely expressed transcripts in each SLE subgroup indicates that specific immunological disease mechanisms are operative in distinct SLE patients’ subsets. This ‘sub-grouping’ approach could further be useful for clinical evaluation of SLE patients and devising targeted therapeutics.

## Introduction

Systemic lupus erythematosus (SLE) is a complex autoimmune disease with diverse presentations of clinical manifestations [1] and wide range of autoantibodies [2]. The major classes of the autoantibody population are targeted against either dsDNA or RNA associated proteins (also known as extractable nuclear antigens, ENA) like Sm, RNP, SS-A, SS-B etc. The tremendous heterogeneity complicates the exact underlying disease mechanisms in SLE that are least understood. Although DNA microarray based studies did contribute to the understanding of SLE [3–7] however, the information was limited to the level of gene expression, SNPs etc. Recent emergence of deep sequencing technology has added newer dimensions in unraveling the disease specific events. These tools have allowed identification of novel transcripts, alternative splicing events and information on non coding RNAs (ncRNAs) associated with SLE [8–10]. In addition, this approach was further employed for identifying rare or novel deleterious variants as genetic causes of SLE [11, 12].

Targeting heterogeneity in SLE is the most challenging aspect of the disease, the underlying cause of which if resolved could pave way for development of personalized or precise treatment in SLE. We in our lab have tried to address heterogeneity in autoantibody responses by studying SLE patients in different groups segregated based on distinct autoantibody specificities. Earlier with this approach we have been able to document that differential expression of toll like receptors (TLRs) [13], heat shock proteins (HSPs) [14], miRNA based genetic regulation [15] and divergent sources of autoantigens [16] exist in different autoantibody subsets of SLE patients.

The deep sequencing approaches used so far in earlier studies have identified gene expression profile in unsegregated SLE patients. We hypothesised based on our previous results that the transcriptomics in different subsets of SLE patient grouped on the basis of autoantibody specificities would be differential and could reveal discrete biological pathways operative in the distinctive subsets. Therefore, in this study, as described earlier [13–16], the SLE patients were characterized into different subsets based on their autoantibody profiles (subset I: anti-dsDNA^+^ or subset II: anti-ENA^+^ or subset III: anti-dsDNA^+^ENA^+^) to identify novel expression patterns of transcripts that would otherwise be missed when studying SLE patients as one common group.

We identified various categories of transcripts like coding, non-coding, antisense, pseudogenes etc. and immunoglobulin (Ig) transcripts using RNA-seq technology that express differentially in SLE patients’ subsets. The expression of coding RNA and Ig varies significantly between different subsets of SLE patients though no significant difference was observed for ncRNAs among them. Further, using Ingenuity pathway analysis (IPA) tool we have identified multiple *cytokine signaling* pathways to be dysregulated in anti-dsDNA^+^ patients whereas *Interferon* (*IFN) signaling* was dysregulated in anti-ENA^+^ patients. IPA analysis of transcripts dysregulated in subset with both anti-dsDNA and anti-ENA autoantibodies shows *regulation of actin based motility by Rho signaling* pathway to be most affected. In addition, different transcripts of same genes were observed to be expressed differentially in each SLE subset. Granulocyte signature genes though present in all SLE subsets had unique distribution of specific transcripts in different subsets. Plasma cell (PC) signature transcripts including the hyper mutated Ig gene transcripts were observed to be differentially distributed in each SLE patient subsets.

Taken together, the identification of distinguishing genetic patterns, transcripts and subset specific events is suggestive of distinct disease driven processes in serologically defined SLE patients. The ‘sub-grouping’ approach employed in this study should therefore prove useful for delineating the diverse disease pathways and developing greater in-depth understanding of SLE.

## Materials and Methods

### Subjects

A total of twenty-eight SLE patients following American College of Rheumatology 1997 revised criteria were recruited from outpatient department at Sir Sunderlal Hospital, Banaras Hindu University (BHU), Varanasi from August 2009 to February 2013. Informed written consent was obtained from all participants enrolled in this study and in case of minors/children (below 18yrs) informed and written consent was taken from their guardians. Institutional ethics committee, Faculty of Science, BHU had approved the study protocol and subject consent. The study was performed in accordance with the 1964 declaration of Helsinki and its later amendments. All patients were female (median age: 30 years, range: 16–45 years) and most of them were on medications generally including prednisolone, hydroxychloroquine, and non-steroidal anti-inflammatory drugs (Table 1). The disease activity of SLE patients was scored using the systemic lupus erythematosus disease activity index-2000 (SLEDAI-2000) [17]. The descriptive clinical features and SLEDAI-2000 scores of SLE patients are presented in Table 1. Twelve age and sex-matched healthy individuals (median age: 28 years, range: 21–43 years, and all females) were enrolled in the study as controls. 5ml of peripheral blood was collected in sterilized heparin-coated tubes and 3ml of unheparinised blood was collected in separate tube. For serum separation blood was allowed to coagulate at RT for 15–20 min followed by centrifugation at 2000 rpm for 10 min and stored at -80°C, deep freezer until use.

**Table 1:** Clinical profile of SLE patients.

Patients I.D.	Age (years)	Anti-dsDNA	Anti-ENA	Clinical Manifestations	SLEDAI-2000 (score)	Medications
*S01*	35	+	-	Glomerulonephritis, Pericarditis, Hepatomegaly	9	Carvidilol, Ramipril, Lasilactone
**S02**	28	+	-	Arthritis, Cutaneous	8	NSAID
***S03***	32	+	-	Pleuritics, Arthritis, Cutaneous, Oral Ulcer	31	Prednisolone, HCQ
*S04*	28	+	-	Glomerulonephritis, Leucopenia, Anemia	15	Prednisolone
*S05*	22	+	-	Myositis	6	Prednisolone, HCQ
***S06***	45	+	-	Arthritis, Cutaneous	11	NSAID
*S07*	17	+	-	Arthritis, Oral ulcer, Cutaneous	18	Prednisolone
*S08*	40	+	-	Glomerulonephritis, Arthritis	12	Prednisolone, HCQ
***S09***	16	+	-	Arthritis, Cutaneous, Oral Ulcer, Thrombocytopenia	12	Prednisolone
*S10*	36	+	-	Oral Ulcer, Cutaneous, Arthritis	12	Phentermine
*S11*	25	+	+	Glomerulonephritis, Arthritis, Oral Ulcer	12	Prednisolone, NSAID
**S12**	36	+	+	Arthritis	8	Prednisolone, HCQ
*S13*	28	+	+	Glomerulonephritis, Anemia	8	Prednisolone
***S14***	32	+	+	Arthritis, Cutaneous	9	Prednisolone
***S15***	20	+	+	Arthritis, Cutaneous	8	Prednisolone
***S16***	27	+	+	Arthritis, Oral ulcer, Cutaneous	13	Prednisolone
*S17*	24	+	+	Glomerulonephritis	6	NSAID, Nefedipine
***S18***	24	-	+	Arthritis, Cutaneous, Oral ulcers	8	Alfacalcidol, Cosval PC 28
**S19**	32	-	+	Arthritis, Cutaneous, Oral ulcer	12	Fexofenadine
*S20*	28	-	+	Oral ulcers, Cutaneous, Leucopoenia	5	Prednisolone
**S21**	36	-	+	Arthritis, Cutaneous, Oral ulcer, Anemia	11	Prednisolone
*S22*	42	-	+	Arthritis, Cutaneous, Oral ulcer	9	Prednisolone, HCQ
*S23*	32	-	+	Myositis, Arthritis	8	Prednisolone, HCQ
*S24*	36	-	+	Arthritis, Cutaneous	6	Prednisolone
**S25**	26	-	+	Pericarditis, Arthritis	14	Prednisolone
*S26*	32	-	+	Glomerulonephritis, Arthritis, Cutaneous, Leucopenia	30	Prednisolone
*S27*	34	-	+	Neurological symptoms, Arthritis, Cutaneous, Oral ulcers, Leucopenia	35	Prednisolone, NSAID
*S28*	19	-	+	Arthritis	8	Prednisolone, HCQ

The sample IDs in bold font were used for the RNA sequencing

The sample IDs in italics font were used for qPCR validation

The sample IDs in both italics and bold font were used for both RNA sequencing and qPCR validation

All patients were female

HCQ Hydroxychloroquine, NSAIDs Non-steroidal anti-inflammatory drugs

### Anti-dsDNA and anti-ENA autoantibodies detection by ELISA

Autoantibodies against dsDNA and ENA were detected in the sera of SLE patients using indirect ELISA kits (Aesku diagnostics, Wendelsheim, Germany). Six ENA antigens (Sm, RNP, SS-A, SS-B, Jo-1 and Scl-60) were pre-coated on the wells of anti-ENA ELISA kit. Standards and patients sera were diluted at the ratio 1:101 as per the manufacturer’s instructions and added to the wells coated with dsDNA or ENA antigens, and incubated for 30 min at RT. Unbound fractions were washed off by washing thrice with wash buffer. Further, the anti-human-IgG conjugated to HRP was added to each well and incubated for 15 min at RT followed by washing, three times with wash buffer. TMB-substrate was added and incubated for 15 min until blue colour appeared. The reaction was stopped by adding stop solution to each well and signals were detected by measuring absorbance at 450 nm wavelength using an ELISA reader (Bio-Rad Laboratories, Hercules, CA).

### Study Groups

SLE patients were categorized into three subsets based on their serum autoantibody profile as determined by indirect ELISA. Subset I included ten patients (S01-S10) positive for autoantibodies against dsDNA only (anti-dsDNA^+^); subset II comprised of eleven patients (S18-S28) positive for anti-ENA autoantibodies only (anti-ENA^+^) and subset III included seven patients (S11-S17) possessing autoantibodies against both dsDNA and ENA (anti-dsDNA^+^ENA^+^).

### Peripheral Blood Leukocyte separation (PBLs) and RNA isolation

Heparinized blood was processed for PBLs separation and RNA isolation. Whole blood was lysed in four volume of RBCs lysis buffer (155 mM NH_4_Cl, 12 mM NaHCO_3_, 0.1 mM EDTA, pH 7.4) at room temperature (RT) and erythrocytes were removed by washing with phosphate buffered saline (PBS). Leukocytes thus obtained lysed in TRI reagent (Sigma-Aldrich, St Louis, MO) for RNA isolation as per the manufacturer’s protocol. Briefly, lysate mixed with the chloroform, centrifuged and the aqueous layer was separated and collected in a fresh tube. Further, RNA in the aqueous layer is precipitated by isopropanol followed by washing with 70% ethanol. RNA samples were treated with DNase to remove the genomic DNA contamination. Their quality was assessed using 2100 Bioanalyzer (Agilent Technologies). Samples with RNA integrity number (RIN) >7 were processed for library preparation and RNA-sequencing.

### Library preparation and RNA sequencing

RNA-seq was performed for a total of sixteen samples including four controls and twelve SLE patient samples; four samples in each patients groups. The patients samples used for RNA-sequencing were marked in Table 1. cDNA library was prepared using Illumina® TruSeq® RNA sample preparation kit as per manufacturer’s instruction. In brief, mRNA molecules were purified using oligo-dT attached magnetic beads and fragmented using divalent cations under elevated temperature. The first strand cDNA strand was synthesized from cleaved RNA fragments using reverse transcriptsase and random primers followed by second strand cDNA synthesis using DNA Polymerase I. RNase H was used to specifically digest the template mRNA. After the end repair process and adenylation of 3ʹ end tailing, adapters were ligated to the cDNA. Samples were then purified and enriched with PCR to create the final cDNA library. Quality of the cDNA libraries were accessed using Agilent Technologies 2100 Bioanalyzer. The libraries were hybridized to the flow cell and cluster was generated by bridge amplification. Paired end sequencing was performed using Illumina® Hiseq-2000 platform.

### Bioinformatics analysis

Output files in fastq format were processed for pre-alignment QC. On an average, ~84% of the total reads of all samples passed ≥ 30 Phred score. Low quality base were trimmed from the reads. Further refinements for the removal of the unwanted sequences including mitochondrial genome sequence, ribosomal RNAs, transfer RNAs, adapter sequences and others were done using bowtie2 (version 2.1.0), tool. The pre-processed reads were aligned to the reference human genome and gene model downloaded from Ensembl database (http://www.ensembl.org/info/data/ftp/index.html) using Tophat program (version 2.0.8) with default parameters. Reads uniquely mapped were considered for further analysis (S1 Table). Cufflink program (version 2.0.2) was used to determine differentially expressed transcripts and genes. To check the reliability and the comparability of differential expression analysis, the transcripts/ genes with FPKM ≥ 1 in all individually sequenced patients and controls were examined. Correlation analysis of differentially expressed transcripts/ genes among the biological replicates was also performed to rule out the possibility of variation among the samples in the group (S1 Fig). A difference of at-least two fold in the transcripts/ genes expression between different subgroups and control were considered for further analysis. Student’s t-test and Benjamini-Hochberg false discovery rate tests were performed for each of the differentially expressed transcripts across the biological replicates. Further, we used DESeq program, another tool to analyze the differentially expressed genes (DEGs) and to compare the results of Cufflink analysis. DESeq is the count based matrices that identifies DEGs only whereas, Cufflink adopts an algorithm that controls cross-replicate variability and read-mapping ambiguity by using a model for fragment counts (FPKM) based on a beta negative binomial distribution that identifies both differentially expressed transcripts and genes. We compared the DEGs results from Cufflink and DESeq analyses, and took the intersection of them for downstream pathway analysis. The datasets from this study have been deposited in the Gene Expression Omnibus repository (GEO series accession number: GSE80183).

### Principal component analysis and Functional analysis

Unsupervised analysis using, principal component analysis (PCA) and hierarchical clustering was performed to visualize the similarities and the distinction between samples belonging to different SLE subsets. Principal component analysis is a mathematical algorithm that extracts important variables (in form of components PC1 and PC2) from a large set of variables available in a data set. The transcripts which showed median FPKM ≥ 1 in all patient samples were used to generate the PCA plot. Further, extensive analysis was performed to identify the relevant bio-functions and the pathways associated with differentially expressed gene transcripts in different subsets of SLE patients’ by using ingenuity pathway analysis (IPA) software (Ingenuity Systems, Redwood City, CA). Differentially expressed (upregulated or downregulated) gene transcripts in each SLE patients’ subsets that shows a minimum of two fold change as compared to that of healthy individuals were imported to IPA for the analysis. IPA generates the pathway utilizing the genes from our dataset referred as ‘focus gene’ and genes stored in ingenuity knowledge database (based on the functional annotation and experimental observation). It also computes a p value for each pathway which indicates the likelihood of the association between focus genes and canonical pathway is not random. A cut-off of p value was set at less than 0.05 (or score > 1.3 score = -lop *P*) and was calculated using Fisher’s exact test to define the significance of the pathways associated with our dataset. Moreover, IPA analysis of overall dysregulated transcripts (upregulated and downregulated; both together) were analysed for the prediction of activated or inhibited canonical pathway based on z-score. IPA automatically calculates the z-score based on differentially expressed genes/ transcripts in our dataset and the information stored in IPA knowledge database. Positive z-score suggests the activation, whereas negative z-score indicates inactivation of the pathway. The pathway with the highest scores and focus molecules were identified by IPA analysis and displayed graphically as a collection of nodes (gene transcripts) and edges (the biological relationships between the nodes). The node color indicates the up-regulation (orange) or downregulation (green) of gene transcripts. In addition to IPA analysis, other gene enrichment approaches were used for the functional characterization of differentially expressed transcripts in distinct SLE subgroups which includes Gene Set Enrichment Analysis (GSEA) and Database for Annotation, Visualization and Integrated Discovery (DAVID) Bioinformatics Database.

### Real-time reverse transcription-polymerase chain reaction (RT-PCR)

The uniquely expressed transcripts identified by RNA-seq analysis in distinct SLE patients’ subsets were further validated using real time PCR. A total of twenty three SLE samples and eight control samples were used for the validation experiments that also includes the samples used for RNA-seq. The patient samples used for qPCR were specifically marked in Table 1. The qPCR was performed using TaqMan® assays (Applied Biosystems, Foster City, CA). CCL20, CCNA1, EPHB2 and ELANE transcripts were selected for validation based on their expression in specific SLE subgroup. CCL20 expressed uniquely in anti-dsDNA^+^, CCNA1 in anti-ENA^+^, EPHB2 in anti-dsDNA^+^ENA^+^ and ELANE in all SLE patient subsets. GAPDH was used as internal control. cDNA was synthesized using High capacity reverse transcription kit (Applied biosystems, Foster City, CA) following manufacturer’s instruction. Briefly, prior to reverse transcription reaction, 2 μg of RNA was subjected to DNase (NEB) treatment to remove the contaminating genomic DNA. Further, reaction mixture comprising DNase treated RNA, RT Buffer (10×), Random Primer (10×), dNTP mix (25×), Reverse transcriptase enzyme, and RNase Inhibitor (10 U/μl) were incubated at 25°C for 10 min followed by incubation at 37°C for 120 min. Finally, enzyme is inactivated by incubation at 85°C for 5 min. Whole reaction was carried out in PCR Thermocycler (Applied biosystems, Foster City, CA). A TaqMan® assay for each gene was used for performing quantitative real time PCR as per manufacturer’s instruction. PCR reactions were carried out on Applied Biosystems 7500 real-time PCR system, using 2 × TaqMan® universal PCR master mixes (Applied Biosystems, Foster City, CA). The comparative Ct method was used to interpret the data as described by Livak and Schmittgen [18]. Relative expression of each gene among SLE patients and healthy individual was determined using formula, Fold change = 2^-ΔΔCt^, where ΔCt = Ct(Gene transcript) − Ct(GAPDH) and ΔΔCt = ΔCt(SLE patient) − ΔCt(Control).

### Statistical Analysis

The statistical analysis of the data was performed using GraphPadprism software v.5.0 (GraphPad Software, San Diego, CA). Statistically significant difference among two or more groups was identified using Kruskal-Wallis H test. The comparison of CCL20, CCNA1, EPHB2 and ELANE gene transcripts expression in each SLE subsets to controls was performed using the non-parametric Mann Whitney test. P-value less than 0.05 were considered as statistically significant.

## Results

### Principal Component Analysis

**Fig 1:**
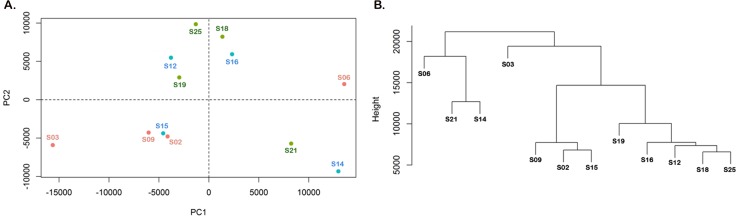
Unsupervised analysis of individual samples that belongs to distinct SLE patients’ subsets. A. Principal component analysis of each SLE patients. Individual dot on scatter pot represent specific SLE patient that were spatially separated based on their transcripts rather than expression values. Red dots represent anti-dsDNA^+^ Group; Green dots belong to anti-ENA^+^ Group and Blue dots belong to anti-dsDNA^+^ENA^+^ Group. B. Dendrogram derived from a hierarchical clustering analysis represents the similarity and distinction among the samples based on distance between datasets (represented as the height of the branches).

The aim of this study was to identify transcriptomic signature in different subsets of SLE patients categorized on the basis of autoantibody profile; Subset I: anti-dsDNA^+^ (patients possessing autoantibody against dsDNA); Subset II: anti-ENA^+^ (patients possessing autoantibody against ENA) and Subset III: anti-dsDNA^+^ENA^+^ (patients possessing autoantibodies both against dsDNA and ENA). Initially, we performed an unsupervised principal component analysis to identify subset specific phenotypes that is more likely to be represented as a function of all transcripts rather than the separate expression values on PCA plot. The PCA analysis was conducted using the transcripts that showed FPKM≥1. Using RNA-seq we identified that anti-dsDNA^+^ patients expressed 36464 transcripts with FPKM≥1 whereas anti-ENA^+^ and anti-dsDNA^+^ENA^+^ patients expressed 34689 and 33703 transcripts with FPKM≥1 respectively. The plot represents a total of 33254 transcripts on PCA plot. The PCA analysis represented individual samples on two principal components (Fig 1A). PCA reveals that samples of anti-dsDNA^+^ subgroup were spatially separated from the anti-ENA^+^ patient samples. Majority of the samples belonging to anti-dsDNA^+^ and anti-ENA^+^ subgroup clustered in their respective class. Anti-dsDNA^+^ENA^+^ patient samples were observed to lie either close to anti-dsDNA^+^ patients or anti-ENA^+^ patients. However, one sample from each subgroup exhibit variation from their respective SLE subgroup. Similar results were observed with hierarchical cluster analysis (HCA) which represents similarity and distinction among the samples. The Dendrogram derived from HCA is based on the Euclidean distance between datasets in the space of the first two PCs which is represented as the height of the branches (Fig 1B).

### Transcriptome Characterization

**Fig 2:**
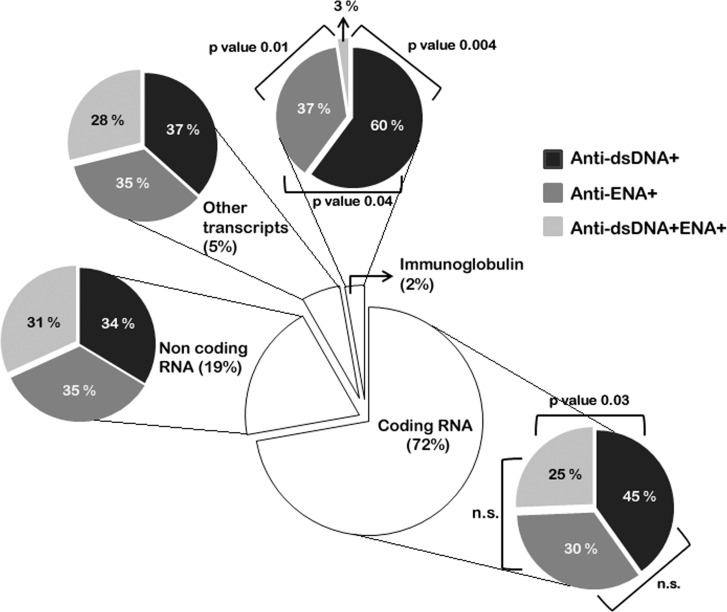
Transcriptome characterization in different SLE patients’ subsets. The pie chart at the centre represents the percentage of coding RNA, non-coding RNA, Ig transcripts and other transcripts (pseudogenes, antisense transcripts, processed transcripts etc.) in SLE patients compared to healthy individuals. Each transcript types was further analysed for each subset of SLE patients. The percentage of coding RNA and Ig transcripts vary significantly in distinct subsets whereas the expression of non-coding RNA and other transcripts was comparable among different subgroups.

After we observed the separation of anti-dsDNA^+^ and anti-ENA^+^ SLE subgroups using unsupervised cluster analysis, it was further interesting to identify the distribution of different class of transcriptome in distinct subsets of SLE patients and their functional relevance. We identified various types of differentially expressed transcripts belonging to different RNA categories in all SLE patient subsets. Upon analysing the datasets we observed protein-coding RNAs constitutes the major class of RNA transcript (3196 transcripts, 72%) compared to other class of RNA like ncRNAs (862 transcripts, 19%), others (antisense transcripts, processed transcripts, pseudogenes etc. (243 transcripts, 5%) and Ig transcripts (123 transcripts, 2%) (Fig 2, S2 Table).We observed that the percentage of dysregulated protein-coding transcripts was not uniform in different subsets of SLE patients, with minimal in anti-dsDNA^+^ENA^+^ subgroups (25%) and maximal in anti-dsDNA^+^ subgroup (45%) (Fig 2). SLE patients represent a diverse array of autoantibodies against self-antigens so we also evaluated the expression of Ig genes in different SLE patients’ subsets. We observed striking difference in the Ig gene transcripts among distinct subsets. Highest percentage (60%) of Ig gene transcripts was observed to be expressed in anti-dsDNA^+^ patients followed by anti-ENA^+^ patients (37%) (Fig 2). There was strikingly reduced number of Ig gene transcripts (3%) in anti-dsDNA^+^ENA^+^ SLE patients (Fig 2). It has been recently identified that ncRNAs are key regulatory molecules for the post-transcriptional modulation of genes [19] and other transcripts, we observed equivalent percentage of ncRNAs and other transcripts expressed in each SLE patients’ subsets (range 31–35% and 28–37% respectively) (Fig 2). Furthermore, different classes of ncRNA species like lincRNA, miRNA snRNA, snoRNA, misc RNA were observed to be dysregulated in all subset of SLE patients (S2 Fig), with no significant difference in their expression among different SLE subsets.

### Differentially expressed transcripts in distinct subsets of SLE patients

**Fig 3:**
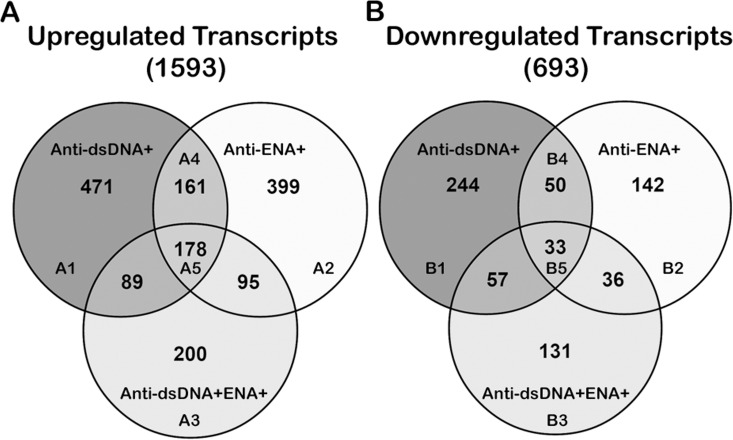
Comparison of dysregulated coding RNAs in distinct SLE patients’ subsets. The venn diagram represents the unique or overlapping coding RNAs that are transcribed in SLE patient with distinct autoantibody specificities. A. Upregulated transcripts and B. Downregulated transcripts in each SLE patient subsets.

Analysis of coding RNA transcripts revealed a total of 2286 transcripts dysregulated (≥ 2 fold) in SLE patients, however it was interesting to note that upregulation of transcript expression (1593 upregulated transcripts) was a predominant event as compared to downregulation (693 downregulated transcripts). Upon further analysis distinct differences in transcripts expressions were observed in different autoantibody subsets. A set of 471 transcripts that were uniquely upregulated in anti-dsDNA^+^ subset; 399 and 200 transcripts were specifically expressed in anti-ENA^+^ and anti-dsDNA^+^ENA^+^ subsets, respectively (Fig 3A, S2 Table). Similar observations were made in the set of down regulated transcripts wherein 244, 142, and 131 transcripts were uniquely downregulated in anti-dsDNA^+^, anti-ENA^+^ and anti-dsDNA^+^ENA^+^ subsets, respectively (Fig 3B, S2 Table). In addition there were 211 commonly dysregulated transcripts (161 elevated and 50 downregulated transcripts) shared by both anti-dsDNA^+^ and anti-ENA^+^ SLE patients; whereas 178 transcripts with increased expression and 33 with reduced expression were observed in all subsets of SLE patients (Fig 3A and 3B). Heat map representing the differentially expressed transcripts in each SLE subsets as compared to controls has been provided in supplementary information (S3 Fig). Further, the expression of CCL20 CXCL3, CCNA1, OPLAH, EPHB2 and IFNG transcripts that were observed to be upregulated in anti-dsDNA^+^/ anti-ENA^+^/ anti-dsDNA^+^ENA^+^ patients, as identified by RNA-seq analysis were graphically plotted to check for the variability among the individual samples and subgroups (S4A–S4F Fig).

### Pathway Enrichment Analysis

The biological relevance and the functional characteristic of the uniquely expressed transcripts associated with distinct SLE patients’ subsets were identified by IPA software analysis. IPA analysis of upregulated and downregulated datasets revealed unique canonical pathways in each SLE subgroups (Table 2). After analyzing the up- and down-regulated transcripts independently and selecting the top affected pathways based on p-value we analyzed these transcripts together to study their effect on particular canonical pathways based on z-scores. An important application of the IPA software is that along with the identification of affected canonical pathway it can predict whether a canonical pathway is activated or inactivated based on the z-score algorithm (Table 3). These canonical pathways have been discussed in detail in next section. Functional analysis by GSEA and DAVID pathway revealed similar pathways as identified by IPA which further confirms the pathway signatures specific to each SLE subsets. S3 Table lists the result of the analysis by GSEA and DAVID, including GO term and KEGG pathways.

**Table 2:** Top canonical pathways associated with uniquely expressed transcripts in distinct SLE patients’ subsets.

Upregulated			Downregulated		
Canonical Pathways	Molecules	p value	Canonical Pathways	Molecules	p value
**Subset I: Anti-dsDNA**^**+**^					
Pattern Receptor Recognition of Bacteria and Virus	C1QB, C3, C3RA1, EIF2AK2(PKR), IRF3/7, IRF7, IL6, IL-10, NLRP3(NALP3), PIK3R5(PI3K), PTX3	1.24E-05	Nur77 Signaling in T Lymphocytes	APAF1, PPP3R1, NFATC1, NR4A1, HLA-DMB	1.87E-04
LXR/RXR Activation	CD36, FASN, IL-1A, IL-6, OLR1, SCD1*	2.45E-04	Role of NFAT in Regulation of Immune Response	AKT1, APAF1, CSNK1D, FOS, FCER1A, HLA-DMB, LYN, NFATC1	2.04E-04
Growth Arrest and DNA Damage Inducible 45 Signaling	GADD45A, GADD45G, CCND3, CCNE1	2.70E-04	Neurotrophin/TRK Signaling	AKT1, CREB1, FOS, FRS2, SHC1	4.00E-04
**Subset II: Anti-ENA**^**+**^					
Complement Signaling	CD46 (MCP), CD55 (DAF), CD59, ITGB2*, ITGAX	1.61E-04	Actin Cytoskeleton	c-SRC, BAIAP2, FLNA, MYL6B, PIRI21	5.85E-03
Interferon Signaling	IFITM2, IRF1, OAS1*, MX1*	1.28E-03	**Metabolic pathways:**		
			a. Acetyl CoA Biosynthesis III	ACLY	5.27E-03
			b. Glycine Biosynthesis I	SMHT2	1.05E-02
			c. Methylglyoxal degradation I	HAGH	1.57E-02
Role of PKR in Interferon Induction and Antiviral Response	IRF1, p53, MAP2K6, MKK3/6, TNFRSF1A	2.36E-03	IK Signaling	FLNA, MYL6B, COX2, TNFR	1.69E-02
**Subset III: Anti-dsDNA**^**+**^**ENA**^**+**^					
Antigen Presentation Pathway	CANX, IFNG, HLA-A, HLA-C, NLRC5	4.12E-07	CDK5 Signaling	ADCY3, FOSB, PPP1R12A, PPP1R7	1.20E-03
CTLA4 Signaling in Tc Lymphocytes	AP2A1, AP2M1, MHC-I, PP2A, GRB2	6.12E-06	Cardiac β Adrenergic Signaling	ADCY3, AKAP, PPP1R12A, PPP1R7	3.53E-03
Crosstalk between Dendritic Cells and Natural Killer Cells	IFN-γ, F-actin, MHC-I, PVRL2	1.11E-04	Mitochondrial Dysfunction	COX5B, COX6C, COX7A2, NDUFV1, SNCA	8.51E-03

*More than one transcript of that gene is dysregulated in different subsets

**Table 3:** Top canonical pathways (on the basis of z-score) associated with dysregulated (upregulated and downregulated) transcripts in distinct SLE patient subsets.

Canonical Pathways	Upregulated transcripts	Downregulated transcripts	p value
**Subset I: Anti-dsDNA**^**+**^			
*TNFR Signaling* (z-score -2)	TANK, TNFAIP3	APAF1, c-FOS	8.72E-03
*IL-3 Signaling* (z-score -1.63)	PI3KR5	AKT1, c-FOS, PPP3R1, SHC1, STAT6	1.04E-02
*IL-2 Signaling* (z-score -0.45)	LCK, PI3KR5	AKT1, c-FOS, SHC1	1.21E-02
*IL-4 Signaling* (z-score N/A)	PI3KR5	AKT1, HLA-DMB, HLA-DBQ2, NFATC1, SHC1, STAT6	1.42E-02
*IL-10 Signaling* (z-score N/A)	IL-1, IL-6, IL-10	CD14, c-FOS	3.2E-02
*IL-12 Signaling* (z-score N/A)	IL-10, JMJD6	ALOX15, c-FOS, STAT6	2.84E-03
*IL-6 Signaling* (z-score N/A)	IL-1, IL-6, IL-6ST, PI3KR5	AKT1, c-FOS, CD14, SHC1, TNFR1	3.33E-03
*IL-15 Signaling* (z-score N/A)	Il-6, LCK, PI3KR5	AKT1, SHC1, STAT6	7.35E-03
*IL-17A Signaling* (z-score N/A)	CCL20, CXCL3, IL-6, LCN2, NFkBIZ	c-FOS	1.26E-02
**Subset II: Anti-ENA**^**+**^			
*Interferon Signaling* (z-score 0.45)	IFITM2, IRF1, OAS1, MX1	BAX, IFNAR1	3.82E-05
*p53 Signaling* (z-score -0.38)	BBC3, MDM4, PML, p53	BAX, PMAIP1, PRKDC	2.62E-03
**Subset III: Anti-dsDNA**^**+**^**ENA**^**+**^			
*Regulation of Actin Based Motility by Rho* (z-score 2)	G-ACTIN, ARP2/3, GDIA	MLCP	2.57E-02
*VEGF Signaling* (z-score 2)	ACTN1, ACTB, HIF1A, GRB2	NA	2.66E-02
*Integrin Signaling* (z-score 2)	ACTN1, ACTB, ARP2/3, GRB2	MLCP	3.78E-02

### Pathways associated with uniquely expressed transcripts in distinct SLE subsets

#### Top signaling pathways affected in anti-dsDNA^+^ Subset

Pattern recognition receptor (PRR) signaling, Liver X receptor/ Retinoid X receptor (LXR/RXR) activation and Growth arrest and DNA damage-inducible 45(GADD45) signaling pathways were associated with upregulated transcripts in anti-dsDNA^+^ patients

The upregulated transcripts (471 uniquely expressed; A1 in Fig 3A) analyzed by IPA revealed activation of various canonical pathways (Table 2). The top three canonical pathways were *PRR in recognition of Bacteria and Viruses*, *LXR/RXR activation and GADD45 signaling*. In this study, we observed up-regulation of NALPs (cytoplasmic PRR), PKR, extracellular PRRs (C1, C3, PTX3), IRF7, IL-6, IL-10 transcripts which have essential role in evoking inflammatory response and are involved in *PRR signaling in recognition of Bacteria and Viruses* (S5 Fig). The dysregulated *LXR/RXR pathway* was found to be associated with overexpression of CD36, FASN, IL-1A, IL-6, OLR1 and SCD1 transcripts. In addition, we also observed the elevated expression of GADD45A, GADD45G, CCND3 and CCNE1 transcripts in anti-dsDNA^+^ patients that are involved in *GADD45 signaling* pathway.

Nur77 Signaling in T lymphocytes, Role of Nuclear factor activated T-cells (NFAT) in regulation of immune response and Neurotrophin/ TRK signaling pathways were associated with the downregulated transcripts in anti-dsDNA^+^ patients

IPA analysis of downregulated set of 244 transcripts (B1 in Fig 3B) revealed *Nur77 signaling in T lymphocytes*, *Role of NFAT in regulation of immune response* and *Neurotrophin/ TRK signaling* as top affected canonical pathways (Table 2). Nur77 (NR4A1) gene has an important role in the elimination of self-reactive T-cells in thymus along with transcripts of APAF1, PPP3R1, NFATC1, HLA-DMB genes (S6 Fig). We also observed decreased expression of NFAT (NFATC1), c-Fos, PP3R1, AKT1, CSNK1D, FCER1A, LYN transcripts in our study that are involved in *regulation of immune response* signaling through *NFAT* which could be suggestive of disruption of proper T-cell signaling in anti-dsDNA^+^ patients. Further, *neurotrophin signaling* is affected due to the dysregulation of intermediate signaling molecules like AKT1, CREB1, FRS2 and SHC1.

Multiple cytokine signaling pathways were associated with overall dysregulated transcripts in anti-dsDNA^+^ patients

**Fig 4:**
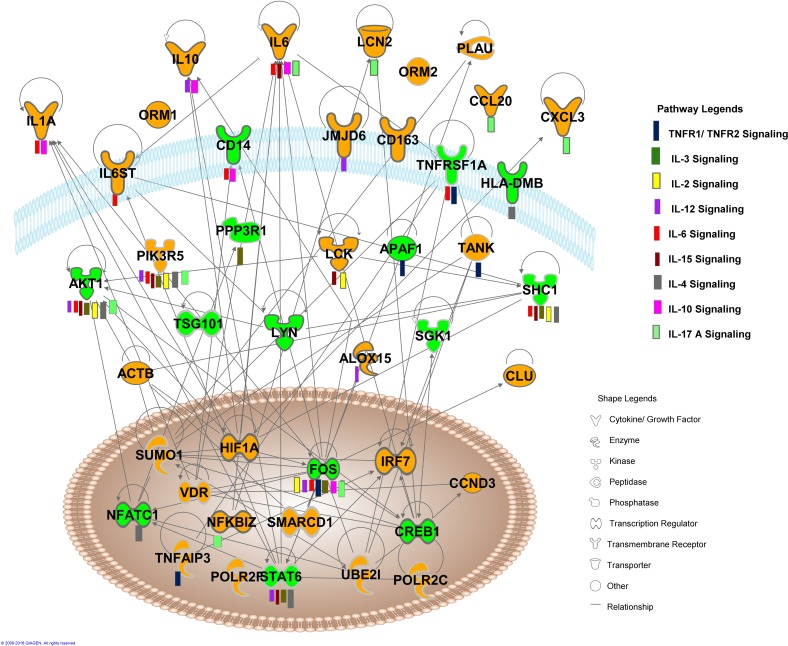
Interactive pathway networks of dysregulated transcripts in anti-dsDNA^+^ SLE patients. The shape legend represents the proteins that are functional as transmembrane receptors, cytokines/growth factors, kinases, peptidases, other enzymes, and transcriptional regulators. The connecting lines indicate direct interactions among the gene transcripts. The pathway legend identifies gene transcripts that were common to the listed pathways affected in the anti-dsDNA^+^ patients. The green nodes in this canonical pathway indicate the downregulated transcripts whereas the orange nodes represent the upregulated transcripts in anti-dsDNA^+^ patients.

Analysis of overall 715 dysregulated transcripts (471 up, A1 + 244 down, B1 in Fig 3A and 3B respectively) in anti-dsDNA^+^ patients, demonstrated dysregulation of various cytokine signaling pathways in anti-dsDNA^+^ patient subsets that included *TNFR signaling*, *IL-3 signaling*, *IL-2 signaling*, *IL-12 signaling*, *IL-6 signaling*, *IL-15 signaling*, *IL-4 signaling*, *IL-17A signaling*, *IL-10 signaling* (Fig 4). The *TNFR signaling* involves dysregulation of TANK, TNFAIP3, APAF1 and cFOS transcripts, where TANK and TNFAIP3 transcripts were upregulated and APAF1 and c-FOS transcripts were downregulated. The dysregulated *IL-3 signaling* was observed to be associated with the reduced expression of c-FOS, SHC1, AKT1, PPP3R1 and STAT6 transcripts and elevated expression of PIK3R5 transcript. Elevated expression of LCK, PIK3R5 transcripts and reduced expression of AKT1, c-FOS, SHC1 transcripts were associated with the dysregulation of *IL-2 signaling*. Further, upregulated transcripts CCL20, CXCL3, IL-1, IL-6, IL-6ST, IL-10, JMJD6, LCK, LCN2, NFkBIZ and PI3KR5 and downregulated transcripts AKT1, ALOX15, c-FOS, CD14, HLA-DMB, HLA-DBQ2, NFATC1, SHC1, STAT6 and TNFR1 were involved in the dysregulation of other cytokine signaling pathways (Table 3). The expression of CCL20 and CXCL3 transcripts that were observed as nodes in interactive pathways of anti-dsDNA^+^ patients derived by IPA tool (Fig 4) were graphically represented for the comparative analysis among individual SLE patient sample of specific subgroup (S4A and S4B Fig).

#### Top signaling pathways affected in anti-ENA^+^ Subset

Complement system signaling, Interferon signaling and Role of PKR in interferon induction and antiviral response pathways were the top affected pathways associated with the upregulated transcripts in anti-ENA^+^ patients

Among the 399 uniquely upregulated transcripts (A2 in Fig 3A) we observed transcripts of CD46, CD55, CD59 genes elevated in anti-ENA^+^ subsets which are complement regulatory molecules. These molecules together with upregulated ITGB2 and ITGAX in anti-ENA^+^ subsets are involved in *complement signaling and regulation* (S7 Fig). Furthermore, antiviral response related signaling like, *Interferon signaling and Role of PKR in interferon induction and antiviral response* were implicated in anti-ENA^+^ patients with the upregulation of IFITM2, IRF1, OAS1, MX1, p53, MAP2K6, MAKK3/6 and TNFRSF1A transcripts in these pathways (Table 2).

Actin cytoskeleton signaling, metabolic pathways and Integrin linked kinase (IK) signaling pathways were the most affected pathways associated with downregulated transcripts in anti-ENA^+^ patients

In contrast to 399 upregulated transcripts, only 142 transcripts were downregulated (B2 in Fig 3B) in anti-ENA^+^ dataset as compared to controls. The top most impacted pathway was *actin cytoskeleton signaling* which plays an important role in cell dynamic processes like motility, cytokinesis and phagocytosis (S8 Fig). Several transcripts like c-SRC, BAIAP2, FLNA, MYL6B, PIR121were downregulated in this pathway. Other transcripts like ACLY, SMHT2 and HAGH that are involved in *metabolic pathways* like *Acetyl CoA biosynthesis III*, *Glycine Biosynthesis I and Methylglyoxal degradation I* pathways, respectively were also seen to have reduced expression. Further, reduced expression of TNFRSF1A, FLNA, MYL6B and COX2 was observed which has potential role in *IK signaling pathway* was also observed to be affected (Table 2).

Interferon signaling and p53 signaling pathways were associated with overall dysregulated transcripts in anti-ENA^+^ subsets

**Fig 5:**
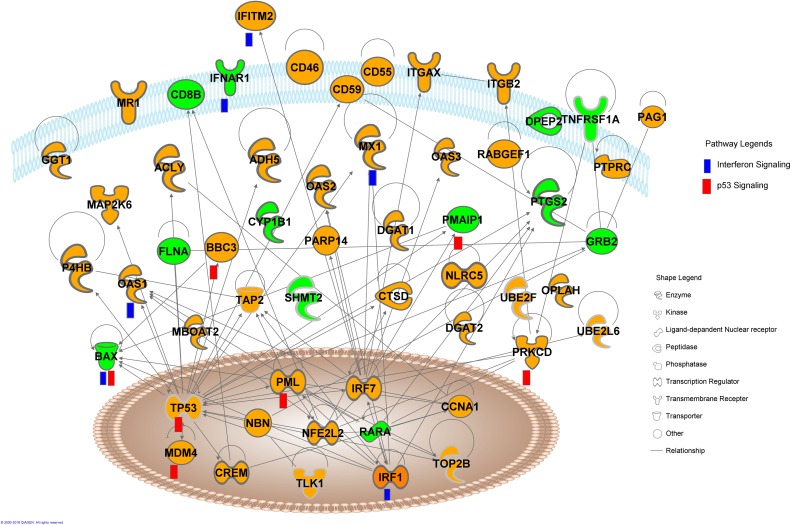
Interactive pathway networks of dysregulated transcripts in anti-ENA^+^ SLE patients. The shape legend represents the proteins that are functional as transmembrane receptors, cytokines/growth factors, kinases, peptidases, other enzymes, and transcriptional regulators. The connecting lines indicate direct interactions among the gene transcripts. The pathway legend identifies gene transcripts that were common to the listed pathways affected in the anti-ENA^+^ patients. The green nodes in this canonical pathway indicate the downregulated transcripts whereas the orange nodes represent the upregulated transcripts in anti-ENA^+^ patients.

Combined IPA analysis of both sets of dysregulated transcripts (399 up, A2 + 142 down,B2 in Fig 3A and 3B respectively) revealed *Interferon signaling* and *p53 signaling* as the top most affected pathway in anti-ENA^+^ patients subset based on z-scores (Fig 5, Table 3). The dysregulated interferon signaling involves downregulated BAX and IFNAR1 transcripts and upregulated IFITM2, IRF1, OAS1and MX1 transcripts specifically. Reduced expression of Bax, PMAIP1 and PRKDC transcripts and elevated expression of BBC3, MDM4, PML and p53 transcripts were associated with dysregulated *p53 signaling*. Further, the expression of CCNA1 and OPLAH, transcripts that were observed to be upregulated in anti-ENA^+^ patients and represented as node in its interactive pathway derived by IPA tool (Fig 5) were plotted to compare the variation among individual samples of specific SLE subgroups (S4C and S4D Fig).

#### Top signaling pathways affected in anti-dsDNA^+^ENA^+^ Subset

Antigen presentation pathway, CTLA4 Signaling in Cytotoxic T-lymphocyte and Cross talk between DC and NK cells are the top affected pathway associated with upregulated transcripts in anti-dsDNA^+^ENA^+^ SLE subset in anti-dsDNA^+^ENA^+^ patients

In anti-dsDNA^+^ENA^+^ patients (A3 in Fig 3A) *antigen presentation pathway*, *CTLA4 Signaling in Cytotoxic T*-*lymphocyte and Cross talk between DC and NK cells* were observed to be affected (Table 2). We in this study observed the transcripts of IFNγ, NLRC5,Class I MHC, CNX (calnexin) genes to be overexpressed in anti-dsDNA^+^ENA^+^ patients that are associated with the processing of typically intracellular or viral proteins eventually presented to CD^+^8 T cells (S9 Fig). Further, in the *CTLA4 Signaling in Cytotoxic T*-*lymphocyte* pathway clathrin adaptor complex like AP2A1 and AP2M1 controls the T-cell activation by endocytosisng CTLA4 molecule followed by its lysosomal degradation. PP2A, MHC-I and GRB2 molecules are the negative signaling proteins involved that interfere with T-cell activation. We observed the upregulation of IFN-γ, F-actin, MHC-I and PVRL2 transcripts which contribute in the crosstalk between NK cells and DCs and have an essential role in immune cell expansion and refinement of the immune response.

CDK5 signaling, Cardiac β-adrenergic signaling and Mitochondrial dysfunction are the top affected pathways in anti-dsDNA^+^ENA^+^ SLE patients associated with downregulated transcripts dataset in anti-dsDNA^+^ENA^+^ patients

IPA analysis of 131 downregulated transcripts (B3 in Fig 3B) demonstrated *CDK5* (Cyclin-dependent kinases) *signaling*, *Cardiac β-adrenergic signaling and Mitochondrial dysfunction* as the most affected pathways (Table 2). Impairment in the *CDK5 signaling* pathway was observed due to the reduced expression of ADCY, PPA1 and FOSB which together may hamper neuronal development, synaptic vesicle trafficking and neurotransmitter release (S10 Fig). Further, ADCY, PPA1 and AKAP also compromise the cardiac contractibility by interfering in the Ca^+2^ ion channels which is crucial for myofilament contraction and relaxation. Most importantly, mitochondrial dysfunction was observed as a result of reduced expression of NDUF1 of complex I, COX5B, COX7A2 of complex IV and SNCA transcripts in anti-dsDNA^+^ENA^+^ subgroups of SLE patients’ specifically.

Regulation of actin based motility by Rho, Vascular endothelial growth factor (VEGF) signaling and Integrin signaling pathways were associated with overall dysregulated transcripts in anti-dsDNA^+^ENA^+^ subset

**Fig 6:**
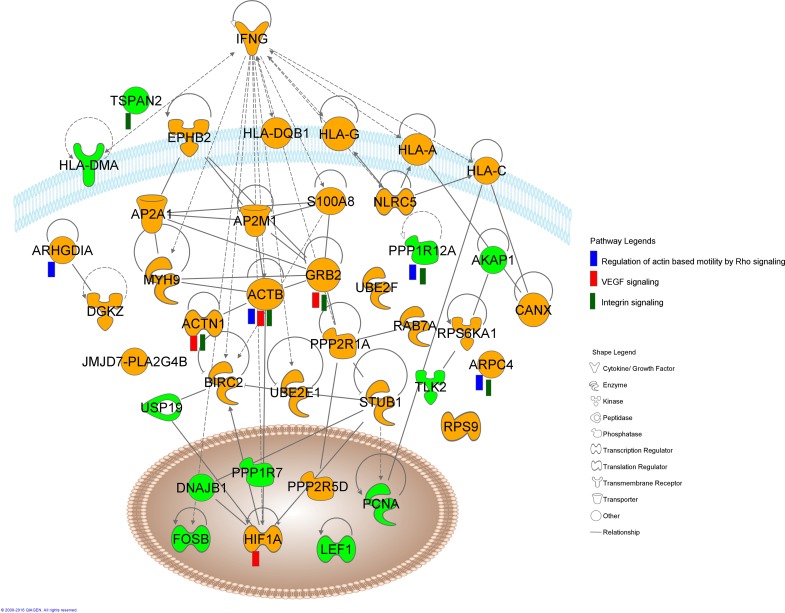
Interactive pathway networks of dysregulated transcripts in anti-dsDNA^+^ENA^+^ SLE patients. The shape legend represents the proteins that are functional as transmembrane receptors, cytokines/growth factors, kinases, peptidases, other enzymes, and transcriptional regulators. The connecting lines indicate direct interactions among the gene transcripts. The pathway legend identifies gene transcripts that were common to the listed pathways affected in the anti-dsDNA^+^ENA^+^ patients. The green nodes in this canonical pathway indicate the downregulated transcripts whereas the orange nodes represent the upregulated transcripts in anti-dsDNA^+^ENA^+^ patients.

The overall dysregulated transcripts (200 up, A3 + 131 down, B3 in Fig 3A and 3B respectively) upon analyzing with IPA revealed *Regulation of actin based motility by Rho*, *VEGF signaling* and *Integrin signaling* as the most affected signaling pathways based on z-score (Fig 6, Table 3). The top most pathway *Regulation of actin based motility by Rho* is associated with dynamic organization of the actin cytoskeleton which provides the force for cell motility and is regulated by small GTPases of the Rho family, in particular Rac1, RhoA and CDC42. They specifically activate several downstream effectors, like G-actin, ARP 2/3, GDIA, which were observed to be upregulated in our study, whereas MLCP was downregulated. Another pathway, *VEGF signaling* observed to be affected in anti-dsDNA^+^ENA^+^ SLE subsets has a fundamental role in growth and differentiation of vascular and lymphatic endothelial cell. We observed elevated expressions of HIF-1α which plays a significant role in inducing transcription of the VEGF gene under hypoxic conditions and it is also responsible for induction of angiogenesis in pathological situations like diabetic retinopathy, tumor angiogenesis and coronary artery disease. Other molecules of this signaling pathway dysregulated in our dataset were α-actinin and actin which has a role in cell migration; GRB2 has a role in cell proliferation. Further, over expression of α-actinin, actin, ARP2/3, GRB2 transcripts and downregulation of MLCP were observed to result into dysregulation of *Integrin signaling* pathway. This pathway has a crucial role in cytoskeleton remodelling and also triggers the activation of mitogen activated protein kinase (MAPK) pathways. Furthermore, expression of EPHB2 and IFNG transcripts in each SLE patient samples were represented graphically to compare the variation among SLE patient sample of specific subgroup (S4E and S4F Fig). These were the differentially expressed transcripts that were observed as nodes in interactive pathway by IPA in anti-dsDNA^+^ENA^+^ (Fig 6).

### Pathways associated with transcripts dysregulated in both anti-dsDNA^+^ and anti-ENA^+^ SLE subgroups or in all SLE patients’ subsets

After analyzing the uniquely expressed transcripts in distinct SLE patient subsets, it was of interest to analyze the common set of transcripts dysregulated in distinct subsets. A total of 211 transcripts (161 up, A4 + 50 down, B4 in Fig 3A and 3B respectively) were dysregulated commonly in anti-dsDNA^+^ and anti-ENA^+^SLE patients’ subgroups. The top affected canonical pathway included *Oncostatin M signaling*, *pancreatic adenocarcinoma signaling* and *VEGF signaling*. Further, the 211 transcripts commonly dysregulated (178 up, A5 + 33 down, B5 in Fig 3A and 3B respectively) in all the three groups were also analysed using IPA which revealed dysregulation of *Citrulline biosynthesis pathway*, *Phagosome maturation pathway* and *IL-8 signaling* pathway (Table 4).

**Table 4:** Canonical pathways associated with transcripts commonly dysregulated among distinct SLE patient subsets.

• Subset I: Anti-dsDNA^+^ • Subset II: Anti-ENA^+^ Subsets		• Subset I: Anti-dsDNA^+^ • Subset II: Anti-ENA^+^ • Subset III: Anti-dsDNA^+^ENA^+^ Subsets	
Canonical Pathways	Molecules	Canonical Pathways	Molecules
*Oncostatin M Signaling*	IL-6ST, JAK, GRB2, MT2A	*Citrulline Biosynthesis*	ARG1, GLS
*Pancreatic Adenocarcinoma Signaling*	TGF-βR1, JAK, GRB2, RALGDS, AKT, MMP9	*Phagosome Maturation*	MHC-I, MPO, RAB7, TBCA
*VEGF Signaling*	HIL-1α, paxillin, AKT, GRB2, α-actinin	*IL-8 Signaling*	CAP3/7, DEFA1, MPO, LIM kinase

### Different transcripts of same genes were differentially expressed in distinct SLE patients’ subsets

**Fig 7:**
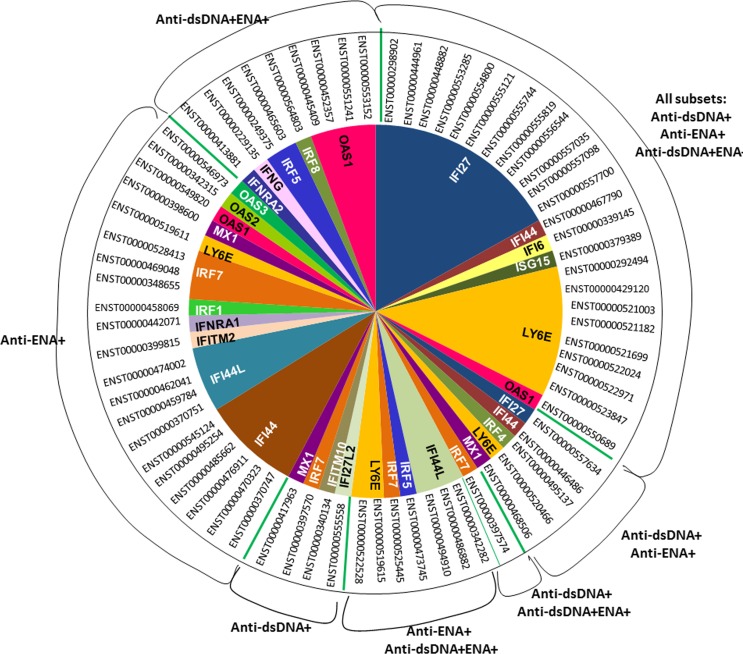
Distribution map of unique or overlapping transcripts expressed in different SLE patient subsets. The circular diagram exhibits distribution of various transcripts of interferon associated genes that are differentially expressed in each SLE subgroup. Ensemble ID in front of each sector represents specific transcript of a gene that is differentially expressed.

It was interesting to observe that various transcripts of the same gene were differentially expressed in each SLE subset. As Interferon and granulocyte gene signatures are of prime importance in SLE we studied the transcripts of the genes related to these signatures in depth. The interferon regulated genes or interferon inducible genes mainly included IRF1, IRF4, IRF5, IRF7, IRF8, IFI6, IFI27, IFI44, IFI44L, ISG15, LY6E, MX1, IFITM2, IFITM10, IFNRA1, IFNRA2, OAS1, OAS2, OAS3 and IFNG. We observed dysregulation of major IFN-inducible gene transcripts in all the three SLE patients’ subsets that included IFI27 (12), LY6E (6), IFI44 (1), IFI6 (1), ISG15 (1), OAS1 (1) (Numbers in parentheses are the count of transcripts of a gene that are differentially expressed) (Fig 7, S4 Table). IFN transcripts were also observed to be uniquely expressed in specific SLE subgroups that are IRF7 (1), IFITM10 (1), IFI27L2 (1), MX1 (1) in anti-dsDNA^+^ subgroup and OAS1 (1), IRF8 (1), IRF5 (1), IFNG (1), IFNRA2 (1) in anti-dsDNA^+^ENA^+^ subgroup (Fig 7, S4 Table). Interestingly, anti-ENA^+^ patient showed relatively large number of dysregulated IFN-related transcripts as compared to other subgroups that includes IFI44 (7), IRF1 (1), IFITM2 (1), IFNAR1 (1), IRF7 (3), OAS2 (1), OAS3 (1) in anti-ENA^+^ (Fig 7, S4 Table). Even though differential distribution of IFNs transcripts were observed in all subsets of SLE patients with comparatively higher number of transcripts dysregulated in anti-ENA^+^ subgroups, it was interesting to observe *IFN signaling* pathway to be affected specifically in anti-ENA^+^ patients as predicted by IPA and GSEA (Tables 2 and 3, S3 Table, S11 Fig).

**Fig 8:**
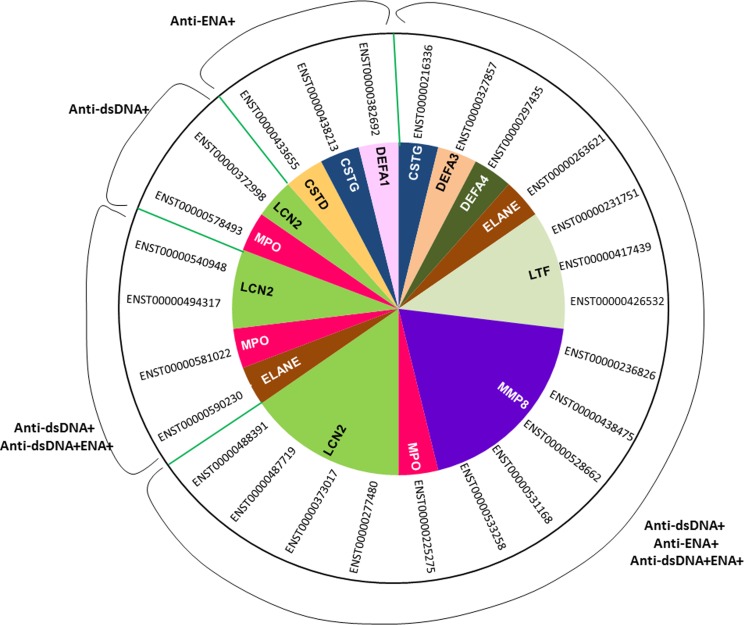
Distribution map of unique or overlapping transcripts expressed in different SLE patient subsets. The circular diagram exhibits distribution of various transcripts of granulocyte associated genes that are differentially expressed in each SLE subgroup. Ensemble ID in front of each sector represents specific transcript of a gene that is differentially expressed.

The dysregulated transcripts belonging to the granulocyte signature genes included transcripts of CSTG, DEFA3, DEFA4, ELANE, LTF, MPO, MMP8, LCN2 and CSTD genes. They too expressed differentially in each subsets with predominant expression (higher fold change) in anti-dsDNA^+^ sub-group and minimal in anti-ENA^+^ sub-group (Fig 8, S5 Table).

### Plasma cell signature transcripts and distribution of Ig gene transcripts in distinct SLE patients’ subsets

SLE is generally characterized by abnormalities in B cell activation which include increased number of circulating Plasma Cells [20] and its frequency has been associated with the production of autoantibodies [21]. Therefore, it was interesting to identify the PC related transcripts and Ig gene transcript distribution in SLE patients segregated on the basis of autoantibody specificities. There are series of molecular events that occurs in mature PCs (Ig secreting long lived cells) which differentiates it from the naïve B cells and plasmablast cells (Ig secreting short lived and proliferative cells). The PCs are characterised by the specific phenotype markers and known expression pattern of cell cycle arrest gene, transcription factors, unfolded protein response (ER stress) and highly mutated immunoglobulin genes [22, 23]. We observed upregulation of PC marker gene transcripts to be mostly associated with anti-dsDNA^+^ and anti-ENA^+^ patients. It mainly includes CD27, CD38, CD43, CD138, GP130 in anti-dsDNA^+^ subgroups and CD27, CD38, CD43, CD44, GP130 in anti-ENA^+^ subgroup whereas, anti-dsDNA^+^ENA^+^ patient show expression of CD38 and CD43 only (Table 5). We also observed dysregulation of transcripts associated with Cell Cycle Arrest/ ER stress (Unfolded protein response)/ Regulatory molecules/ Transcription factors Genes like IRF4, STAT6, ID3, ICSBP (IRF8), CD9, GADD45A, GADD45G, HERPUD1, ERO1L, PDIA3, DNAJC10, DNAJC4, TNFRSF14 (CD270), LAIR1 (CD305), SLAMF7 (CD319), VDR, FYN, FKBP11, BCL11A, HLA-DMB, HLA-DMA and PCNA either in one or more subset of SLE patients (Table 5). Dysregulation of these transcripts were mainly observed in anti-dsDNA^+^ and anti-ENA^+^ patients while comparatively low number of PC related transcripts were expressed in patients with anti-dsDNA^+^ENA^+^. Further, as we mentioned previously the distribution of Ig gene transcripts varies significantly among different subsets of SLE patients (Fig 2). We observed elevated expression of variable regions of heavy and light chain and constant heavy chain region of Ig gene transcripts in all SLE patient subsets with highest distribution in anti-dsDNA^+^ patients than in anti- ENA^+^ patients and lowest in anti-dsDNA^+^ENA^+^ patients (Table 6) which is in concordance with the variation of PC related transcripts in each SLE subsets.

**Table 5:** Plasma cell signature transcripts in each subset of SLE patients.

Ensemble Transcript ID	Gene Name	Fold Change		
		Anti-dsDNA^+^	Anti-ENA^+^	Anti-dsDNA^+^ENA^+^
**Transcripts of Plasma Cell Phenotypic Markers Gene**				
ENST00000541233	CD27	2.66854	2.06851	
ENST00000502843	CD38	3.75347	3.3366	2.08697
ENST00000510674	CD38	3.01971	2.4204	
ENST00000506191	CD38	2.85168		
ENST00000436527	CD43 (SPN)	2.48244	2.4036	2.19705
ENST00000525211	CD44		3.9942	
ENST00000278385	CD44		2.34032	
ENST00000254351	CD138 (SDC1)	4.26323		
ENST00000583149	IL6ST (GP130)	2.12686	2.38951	
ENST00000423954	IL6ST (GP130)	2.03422		
ENST00000503773	IL6ST (GP130)	2.38718		
**Transcripts belonging to Cell Cycle Arrest/ ER stress (Unfolded protein response)/ Regulatory molecules/ Transcription factors Genes**				
ENST00000495137	IRF4	2.48567	2.83529	
ENST00000555318	STAT6	-3.8789		
ENST00000463312	ID3	-2.30003	-2.20641	
ENST00000486541	ID3		-2.15263	
ENST00000564803	ICSBP (IRF8)			-2.07877
ENST00000536586	CD9		2.03671	
ENST00000370985	GADD45A	2.87296		
ENST00000252506	GADD45G	2.01962		
ENST00000570273	HERPUD1	2.52642		
ENST00000554251	ERO1L		2.01659	
ENST00000469684	PDIA3	2.67067		2.98431
ENST00000444005	DNAJC10	2.48372		
ENST00000542376	DNAJC4		2.42485	
ENST00000434817	TNFRSF14 (CD270)	3.73583	3.82959	3.83707
ENST00000391742	LAIR1 (CD305)	2.36958		
ENST00000495334	SLAMF7 (CD319)	2.05426		
ENST00000395324	VDR	14.0071		
ENST00000552878	FKBP11	2.54432		
ENST00000524310	FYN		-2.1386	
ENST00000489516	BCL11A			-2.20888
ENST00000414017	HLA-DMB	-2.32381		
ENST00000475627	HLA-DMA			-2.03516
ENST00000379160	PCNA			-2.24001

**Table 6:** Immunoglobulin gene transcript distribution in different SLE patients’ subsets.

***Immunoglobulin transcripts upregulated in anti-dsDNA***^***+***^ ***patients***		***Immunoglobulin transcripts upregulated in anti-dsDNA***^***+***^ ***patients***		***Immunoglobulin transcripts upregulated in anti-ENA***^***+***^ ***patients***	
Gene Name	Fold Change	Gene Name	Fold Change	Gene Name	Fold Change
IGHE	5.01008	IGLV3-21	2.37023	IGHV3-20	2.69265
IGLV3-25	4.53406	IGKV3D-20	2.36901	IGKV1D-17	2.68896
IGHV3OR16-9	4.22379	IGHV1-2	2.36856	IGHV4-59	2.63234
IGHV3OR15-7	4.16669	IGHV1-24	2.3631	IGKV1D-39	2.56262
IGHV2-26	4.03988	IGHV5-51	2.35946	IGLV3-19	2.52421
IGHG1	3.91708	IGKV1-17	2.35657	IGHV3-64	2.50537
IGLV5-48	3.8949	IGKV3-20	2.35618	IGLV6-57	2.4802
IGHV1-46	3.64612	IGKV3-15	2.34104	IGLV3-21	2.40861
IGHG3	3.55228	IGHV3-7	2.33229	IGKV2D-28	2.39031
IGHG1	3.5107	IGLV3-1	2.32365	IGHV4-28	2.36432
IGHV3-43	3.48571	IGHV3-30	2.29346	IGKV1-17	2.3579
IGLV1-47	3.20425	IGHV3-15	2.28382	IGKV1D-13	2.34903
IGKV2D-29	3.1218	IGLV2-8	2.25405	IGKV2D-29	2.3415
IGLC1	3.11064	IGLV3-19	2.22569	IGLC1	2.3343
IGLV5-37	3.10257	IGLC3	2.21608	IGHV1-2	2.33097
IGLV6-57	3.06899	IGHV2-70	2.21583	IGLV3-10	2.32674
IGHV3-74	3.03379	IGKV2D-28	2.20204	IGHV1-58	2.32128
IGLV2-23	3.0054	IGHV1-3	2.17755	IGLV3-1	2.30196
IGHV3-53	2.94341	IGKV4-1	2.17476	IGKV5-2	2.25375
IGLV3-27	2.92944	IGKV5-2	2.16606	IGLV1-51	2.24319
IGLV1-51	2.91894	IGKC	2.15947	IGHV4-34	2.21088
IGLV4-69	2.90754	IGKV1-5	2.13434	IGLC7	2.20277
IGLV3-10	2.89567	IGHG2	2.13271	IGLV9-49	2.18536
IGKV3D-15	2.86337	IGHV3-9	2.12835	IGKV2D-30	2.1733
IGLV10-54	2.80356	IGHV4-59	2.12121	IGLV5-37	2.15
IGHV3-66	2.72347	IGKV1-12	2.08012	IGKV3D-15	2.14245
IGLV2-11	2.71128	IGHV1-18	2.0192	IGHV1-18	2.12713
IGHV3-64	2.68789	IGHV4-34	2.01819	IGLC2	2.12173
IGHV1-58	2.66096	IGKV3-11	2.00477	IGHV6-1	2.11176
IGLV1-44	2.64481	IGHV3-48	2.00062	IGHV1-8	2.04692
IGHV4-39	2.62203	***Immunoglobulin transcripts upregulated in anti-ENA***^***+***^ ***patients***		IGHV1-3	2.03445
IGLC2	2.61745			IGLV2-8	2.02946
IGLV3-9	2.56444			IGKV1D-16	2.02934
IGLV1-40	2.56427	**Gene Name**	**Fold Change**	IGKV3-15	2.0289
IGHG4	2.55753	IGHV3OR15-7	3.72364	IGKV3D-20	2.02365
IGLV8-61	2.5448	IGHG1	3.38547	IGHV4-39	2.00308
IGHV3-49	2.542	IGHV2-26	3.36045	***Immunoglobulin transcripts upregulated in anti-dsDNA***^***+***^***ENA***^***+***^ ***patients***	
IGKV1-27	2.431	IGHG3	3.30144		
IGLV4-3	2.42931	IGHG1	3.27478		
IGHV2-5	2.42676	IGHV3-43	3.26862	**Gene Name**	**Fold Change**
IGLV3-16	2.41966	IGHV4-4	2.92619	IGHV3-43	2.54058
IGLV2-14	2.39606	IGHV3-66	2.87176	IGLV5-37	2.02747
IGLV9-49	2.39278	IGHG4	2.72909		
IGLV4-60	2.38172	IGLV3-9	2.69396		

### Validation by TaqMan real time RT-PCR

**Fig 9:**
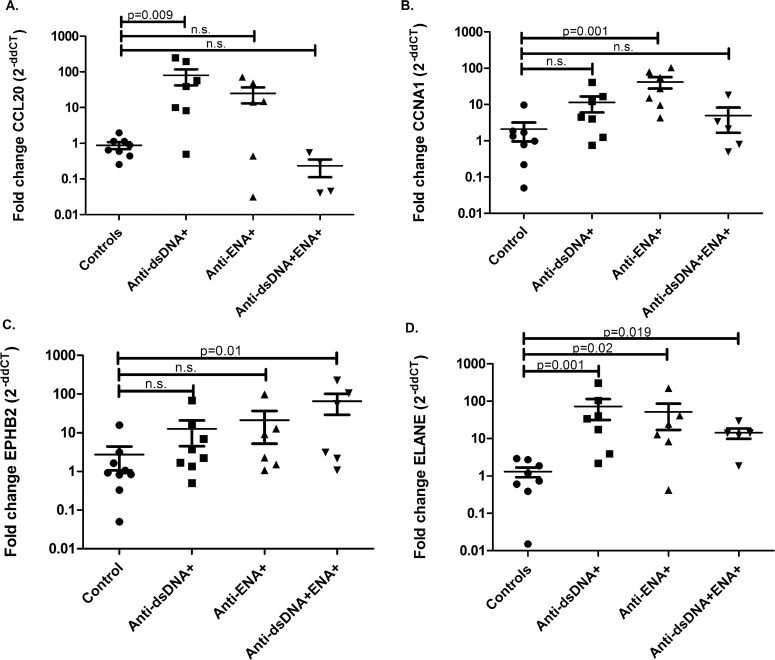
Validation of differentially expressed transcripts in distinct SLE patients’ subsets by real time PCR. A. CCL20 was significantly overexpressed in anti-dsDNA^+^ patients (p value 0.009) B. CCNA1 specifically overexpressed in anti-ENA^+^ patients (p value 0.001) C. EPHB2 expression was observed to be significantly overexpressed in anti-dsDNA^+^ENA^+^ patients (p value 0.01) and D. ELANE was significantly overexpressed in all patient subsets (anti-dsDNA+ patients p value 0.001, anti-ENA^+^ patients p value 0.02 and anti-dsDNA^+^ENA^+^ patients’ p value 0.01) but had higher expression in patients with anti-dsDNA autoantibody.

RNA-seq was performed for a total 16 samples, 4 in each SLE subgroups including healthy individuals. After analysis, four uniquely dysregulated transcripts were selected from each SLE subgroups, CCL20 (upregulated in anti-dsDNA^+^), CCNA1 (upregulated in anti-ENA^+^), EPHB2 (upregulated in anti-dsDNA^+^ENA^+^) and ELANE (upregulated in all subsets) for further validation by TaqMan real time PCR. Further, more samples were included in each SLE subgroups for the validation experiments. We observed that the results obtained by TaqMan real time PCR were in concordance to those observed by RNA-seq experiments (Fig 9). CCL20 was observed to be significantly overexpressed in anti-dsDNA^+^ patients (p value 0.009) (Fig 9A). CCNA1 expression was specifically elevated in anti-ENA^+^ patients (p value 0.001) (Fig 9B). EPHB2 expression was observed to be significantly overexpressed in anti-dsDNA^+^ENA^+^ patients (p value 0.01) (Fig 9C) and ELANE was significantly overexpressed in all patient subsets compared to controls (in anti-dsDNA^+^ patients p value 0.001; anti-ENA^+^ patients p value 0.02 and anti-dsDNA^+^ENA^+^ patients p value 0.01) but had higher expression in patients with anti-dsDNA autoantibody (Fig 9D).

### Differentially expressed genes identified by Cufflink and DESeq analysis

**Fig 10:**
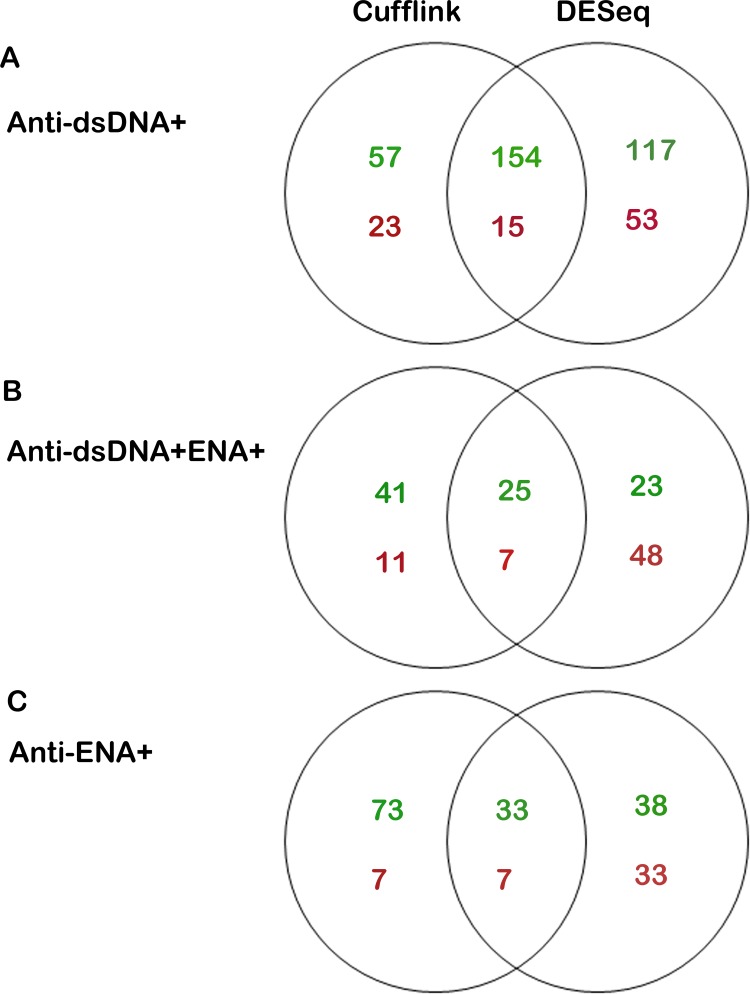
Comparison of differentially expressed genes by Cufflink and DESeq analysis tool. Venn diagram shows the intersection of the DEGs obtained from Cufflink and DESeq analysis and DEGs that were obtained from either Cufflink or DESeq only. Text in green shows number of upregulated DEGs whereas, text in red represents number of DEGs downregulated in each case A. Comparison in anti-dsDNA^+^ B. Comparison in anti-dsDNA^+^ENA^+^ C. Comparison in anti-ENA^+^

**Fig 11:**
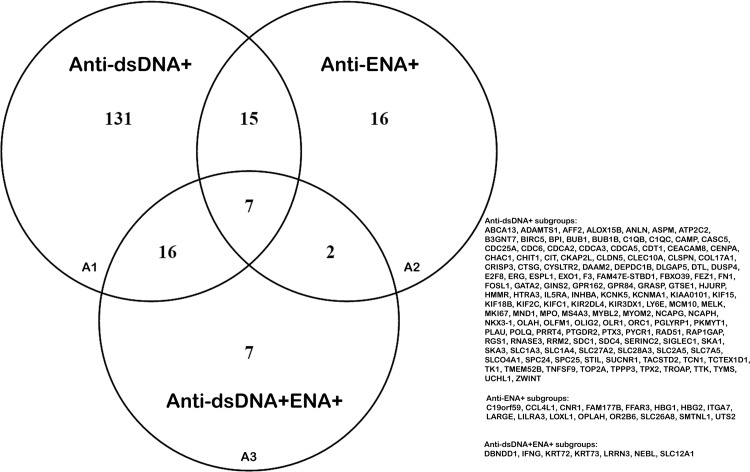
Comparison of DEGs obtained from intersection of Cufflink and DESeq analysis in distinct SLE patients’ subsets. The venn diagram represents the unique or overlapping DEGs in SLE patient with distinct autoantibody specificities.

Differentially expressed (upregulated or downregulated) genes obtained from Cufflink and DESeq analysis tools in each SLE patients’ subsets that show a minimum of two fold changes as compared to that of healthy individuals were used for further analysis. The number of differentially expressed genes derived from DESeq analysis was greater in anti-dsDNA^+^ and anti-dsDNA^+^ENA^+^ subgroups as compared to that of Cufflink tool (Fig 10A and 10B). However, DEGs derived from cufflink analysis was comparatively higher than that of DESeq in anti-ENA^+^ patients (Fig 10C). A total of 169, 40 and 32 genes were commonly observed with Cufflink and DESeq analyses in anti-dsDNA^+^, anti-ENA^+^ and anti-dsDNA^+^ENA^+^ patients, respectively (Fig 10A–10C) (S6 Table). Further, we identified that 131 genes were uniquely expressed in anti-dsDNA^+^ patients (A1 in Fig 11), 16 and 7 unique genes expressed in anti-ENA^+^ and anti-dsDNA^+^ENA^+^ patients respectively (A2 and A3 in Fig 11).

#### Pathway analysis for differentially expressed genes

IPA analysis of the uniquely expressed genes in anti-dsDNA^+^ patients (A1 in Fig 11) revealed the *Cell cycle Control of Chromosomal Replication* as the top affected pathway (p value 8.39E-04) (S12 Fig) which mainly involve like CDC6, CDT1, ORC1, MCM10 and CDC25 genes. Similar *Cell Cycle* pathway was also observed with David pathway analysis (p value 6.4E-04). In anti-ENA^+^ and anti-dsDNA^+^ENA^+^ subsets, pathway information could not be generated by IPA and David owing to small number of dysregulated genes in these subsets (A2 and A3 in Fig 11).

## Discussion

SLE patients are known to exhibit tremendous heterogeneity in clinical presentations. In our earlier studies we have reported that miRNA based immuno-regulatory mechanisms [15], TLR-7 and -9 expressions [13], small HSP involvement [14] and sources of autoantigen pool [16] differentially prevail in different SLE patients’ subsets with distinct autoantibody specificities. These findings clearly points towards the interesting observation that the SLE subset specific disease events could often be missed if studied as a single group. The result of the present study design further builds upon the same concept by clearly indicating that a differential transcriptome profile (genes and transcripts) exists for different groups of SLE patients categorized on the basis of distinct autoantibody specificities. The gene transcripts are the mRNAs that are generated from the same locus either by alternative splicing or alternative promoter usage of a gene. The regulation by different gene transcripts is a critical part of disease processes which is not much explored in SLE. The differentially expressed transcripts among different SLE subsets (identified by Cufflink analysis of RNA-seq data), as observed in this study would have a crucial impact on various biological processes that may result into phenotypic differences among different SLE patients.

An unbiased approach for the transcriptome analysis in SLE patients indicated that patients with anti-dsDNA^+^ and anti-ENA^+^ autoantibodies have specific clinical phenotype that sets them apart. Anti-dsDNA^+^ENA^+^ patients show some similarity with other group of patients which could be due to the presence of autoantibodies against both dsDNA and ENA, which may contribute towards shared phenotype. Though, some samples lie apart from their respective group that could be due associated clinical manifestation in that particular patient. Extensive analysis of distinct SLE sub-groups revealed unique genetic patterns in each subset (SLE patient with anti-dsDNA autoantibody or anti-ENA autoantibody or patient with both autoantibodies). Further, use of IPA analysis predicted the functional relevance of distinctly expressed transcripts which were found to be associated with unique immunological pathways in each SLE subsets. The top most affected pathway in anti-dsDNA^+^ sub-group was *role of pattern recognition receptors in bacteria and viruses* suggesting that the innate immune system which has an important role in the production of pro-inflammatory cytokines are preferentially dysregulated in this subgroup of SLE patients. Interestingly, multiple cytokine signaling pathways were also observed to be dysregulated in anti-dsDNA^+^ patients. Cytokine imbalance is very well known to be implicated in SLE pathogenesis [24] and the levels of various cytokines have been demonstrated to correlate with anti-dsDNA levels in SLE patients [25, 26]. Further, the dysregulation of cytokine signaling specifically in this subset of SLE patients could also be due to miRNA mediated regulation as reported previously [15]. *LXR/RXR pathway* found to be affected in anti-dsDNA^+^ patients is involved in lipid metabolism and inflammation. Recently, a study reported that perturbation in lipid homeostasis in SLE patients affects the functioning of T cells [27]. Other affected pathways are *Nur77 signaling in T lymphocytes* and *Role of NFAT in regulation of immune response*. Both the pathways are central to T cell signaling which is considered to have an important role in SLE pathogenesis [28]. Nur77 which is downregulated in anti-dsDNA^+^ patients is crucial for TCR-mediated thymocyte apoptosis for the elimination of self-reactive TCRs [29]. Persistence of the autoreactive T-lymphocytes further leads to the activation of B-cells and autoantibody generation contributing to the pathogenesis of lupus. Enhanced calcium-calcineurin NFAT signaling has been reported in SLE patients [30] whereas another study has demonstrated decreased calcineurin expression depending upon the glucocorticosteroid [31]. These drugs are generally used for the treatment of SLE [32]. It is important to note here that the patients in each SLE subsets were on immunosuppressive drugs, the difference observed in the expression pattern of transcripts could be the outcome of specific disease driven process. We observed dysregulation of *GADD45 signaling* which is known to have a crucial role in DNA damage and replication. Li et al 2010, have reported that overexpression of GADD45, contributes to SLE pathogenesis by promoting demethylation in T cells [33]. Dysregulation of *Neurotrophin/ TRK signaling* pathway in anti-dsDNA^+^ subgroup as observed in this study have been reported to be associated with T-cell development [34] and neuronal functions [35]. Neurotrophins were reported in neuropsychiatric SLE (NPSLE) patients and its increased expression was associated with improved neuropsychiatric symptoms [36] but no association has been reported in SLE patient with specific serological group.

In contrast to anti-dsDNA^+^ SLE patients, anti-ENA^+^ patients show increased expression of complement regulatory molecule CD46, CD55, CD59 and ITGB2, ITGAX thus affecting *complement signaling*. Elevated CD46 expression had been previously reported in SLE sera [37] but Alegretti et al., 2012 showed diminished expression of CD46, CD55 and CD59 in peripheral blood of SLE patients with haematological involvement [38]. Furthermore, we observed interferon regulated or inducible transcripts are elevated in patients with anti-ENA autoantibody which thus affect *IFN signaling*. Environmental triggers like viral infection are reported to induce IFNs which are central to SLE pathogenesis [39, 40]. Another pathway, *role of PKR in interferon induction and antiviral response*, is affected in the same group of SLE patients involving transcripts of IRF1, TNFRSF1A, p53, MAP2K6 and MKK. Apart from the interferon induction, IRF1 also contributes to the dysregulated epigenome that leads to perpetuation of SLE [41]. These MAPK signaling molecules were observed to be hyperactivated and are shared among various networks in lupus [42]. Molecular mimicry between a particular protein of Epstein-Barr virus (a suspected SLE causing agent) and Sm protein (an anti-ENA target) have been widely recognized as an initial trigger in the development of the autoimmunity. This molecular mimicry could attribute to the dysregulation of viral associated pathway specifically in anti-ENA^+^ patients [43, 44]. *Lipid metabolism*, *amino acid metabolism* and *methylglyoxal degradation pathways* were found to be associated with anti-ENA^+^ patients. Although these pathways are not reported in SLE so far but several other metabolic pathways like glycolysis, Krebs cycle, fatty acid β oxidation and amino acid metabolism have been reported to be defective in SLE patients [45]. Abnormalities in *actin cytoskeleton signaling* and *IK* signaling were observed in anti-ENA^+^ patients in particular. It is speculated that the dysregulation of actin cytoskeleton signaling specifically in anti-ENA^+^ patients results from miRNA mediated regulation as reported by Chauhan et al., 2014 [15]. However, abnormal actin cytoskeleton distribution patterns were reported in bone marrow-derived mesenchymal stem cells of SLE patients compared to healthy controls [46].

In SLE patients with presence of both anti-dsDNA and anti-ENA autoantibodies (anti-dsDNA^+^ENA^+^) it was observed that *Antigen cross presentation pathway* and effector *CTLA4 signaling in T-lymphocytes* pathways were significantly affected. Overexpression of IFN-γ and calnexin in our study is suggestive of increased processing and endosomal trafficking of antigens and presentations to the cytotoxic T-lymphocytes that was reported to be involved in the pathogenesis of lupus nephritis [47]. CTLA-4 is a critical gatekeeper of T-cell activation and immunological tolerance and has been implicated in patients with a variety of autoimmune diseases through genetic association. Abnormal CTLA functioning has also been reported in SLE [48]. The transcripts of the gene involved in *CTLA-4 signaling* like AP2A1, AP2M1, MHC-I, PP2A and GRB2 were observed to be increased in anti-dsDNA^+^ENA^+^ patients. It is thus possible that the over-activation of CTLA-4 signaling may impact other T-cell signaling pathways and contribute to SLE pathogenesis. Impairment of the NK cell function had been reported in SLE patients [49] which could be due to miRNA mediated dysregulation [50, 51]. Aberrant expression of DC cells is also documented in SLE patients [52]. These two cell types together may contribute to the pathogenesis of SLE via cross talk. Further, dysregulation of *CDK5 signaling* was observed in this SLE subgroup. Jeffries et al., 2011 also reported CDK5 signaling pathway to be affected among hypomethylated genes in CD4+ T cells in lupus [53]. Dysregulation of Clathrin-mediated endocytosis and CDK5 signaling had previously been reported in anti-dsDNA^+^ SLE patients due to miRNA mediated regulation [15]. *Mitochondrial dysfunction* was observed to be associated with downregulated transcripts in anti- dsDNA^+^ENA^+^ patients. Mitochondrial dysfunctioning was widely observed in SLE patients which supports our present finding [54, 55]. Moreover, G-actin is an important molecule that is primarily involved in the signaling associated with dynamic organization of actin cytoskeleton [56]. Instead, these are also known to inhibit the Deoxyribonuclease-I (DNase-I) activity [57, 58] which is an endonuclease responsible for degrading DNA from neutrophil extracellular traps (NETs) [59]. Thus, inhibition of the DNase activity also leads to the pathogenesis of SLE [58].

Earlier reports established that IFN signature and granulocyte signature genes are dysregulated in SLE patients [4, 7, 60]. It is important to mention here that these studies were conducted on unsegregated SLE patients whereas a recent study in paediatric lupus patients reported the association of IFN signature and neutrophil signature with specific group of patients. IFN signature was associated with disease activity and neutrophil signature was enriched in active lupus nephritis [61]. However, in the present study, large number of differentially expressed transcripts of IFNs gene family have been preferentially identified in anti-ENA^+^ patients and predominant expression of granulocyte signature gene in anti-dsDNA^+^ patients. It was interesting to note that the interferon alpha pathway was observed to be enriched in the anti-ENA^+^ SLE subsets although specific subtypes of ENA autoantibodies (Sm, RNP, SS-A, SS-B, Jo-1 and Scl-60) were not evaluated in this study. High interferon levels had been shown to be associated with elevated level of anti-dsDNA and anti-ENA autoantibodies in SLE patients [62], however Niewold et al, 2005 reported increased levels of anti-SSA autoantibodies and ANA whereas no change in the anti-dsDNA titre was seen after IFN-α treatment in hepatitis C patient, who developed *de novo* SLE [63]. Another study also reports higher interferon score in SLE patients with ENA autoantibodies whereas no difference was observed in IFN score with variation of anti-dsDNA status [64]. Moreover, majority of neutrophil associated autoantigens that were observed to be enriched in neutrophil extracellular traps (NETs) and are reported to be highly expressed in SLE as compared to other autoimmune diseases such as rheumatoid arthritis, vasculities, multiple sclerosis etc. [65]. The elevated NET formation and their inadequate degradation also contribute to the autoantigen pool in SLE patients [59, 66]. In another study in our lab, we observe that the NET degradation and its clearance are significantly compromised in anti-dsDNA^+^ patients whereas it is comparable to healthy individual controls in anti-ENA^+^ patients [16]. These observations strengthen the view that unique pathological events would be associated with each SLE patients’ subset with distinct autoantibody specificity. We therefore propose that SLE groups to be studied in segregation so that precise clinical evaluation and treatment pertaining to specific groups should be devised.

Our observation of markedly large number of dysregulated PC related transcripts including Ig gene transcripts in anti-dsDNA^+^ and anti-ENA^+^ patients as compared to anti-dsDNA^+^ENA^+^ was interesting. Moreover, the frequency of circulating plasma cell had been reported to be associated with anti-dsDNA titres in SLE patients [21]. In contrast, high titres of anti-Sm/RNP and Ro/La autoantibodies had been shown to be associated with long-lived plasma cells whereas plasmablast cells, which are more susceptible to immunosuppresive or targeted B cell therapies, are responsible for the production of anti-dsDNA antibodies [67, 68]. It is, therefore, possible that the production of specific autoantibodies against dsDNA or ENA may result from other immuno-regulatory disturbances such as dysregulation of TLRs [13, 69, 70]. despite of similar expression pattern of PC related transcripts in anti-dsDNA^+^ and anti-ENA^+^ patients as observed in this study.

In addition, the transcripts like IL-6ST, TGFβ-R1, JAK, AKT, GRB2, MT2A, RALGDS, were observed to be commonly elevated in anti-dsDNA^+^ and anti-ENA^+^ SLE patients. Previous studies had shown their association with the inflammation [71–73], cell proliferation and growth signaling [74, 75] in SLE patients. However, Inhibition of JAK-STAT signaling and PI3/AKT/mTOR signaling had been suggested to be a potential treatment option for SLE [76, 77]. RALGDS was reported in SLE PBMCs whereas MT2A and GRB2 were observed to be upregulated in lupus T cells [78, 79]. MT2A has been reported to play crucial role in leukocyte chemotaxis [80].

Besides observing the uniquely expressed transcripts in patients with different autoantibody specificity, we have also identified transcripts that were commonly dysregulated in all three subsets. ARG1, GLS are the important enzymes involved in the *citrulline biosynthesis*. The antibodies against cyclic citrullinated proteins (CCP) are serological biomarker for rheumatoid arthritis whereas 10–15% of SLE patients also exhibit anti-CCP autoantibodies [81]. Rab7 that was found to be elevated in all subsets of SLE patients which plays a crucial role in regulating membrane traffic between the endo/lysosomal system and *phagosome maturation* at the time of internalization of pathogens or apoptotic cells [82]. It is also evident that the phagocytosis efficiency of the neutrophils and macrophages are compromised in SLE patients [83, 84]. Furthermore, elevated expression of CAP3/7, DEFA1, MPO and LIM kinase transcripts was observed in all SLE patients’ subset that are involved in *IL-8 signaling*. IL-8 signaling is associated with neutrophil migration and activation as a result of inflammation [85].

In-depth analysis of DEGs using two different softwares were performed because there is no general consensus regarding the best analysis tool for the differential expression analysis of RNA-seq data [86, 87], DESeq program was also used to support the Cufflink analysis. The functional analysis of commonly expressed genes, as identified by both Cufflink and DESeq software in anti-dsDNA^+^ patients shows involvement of *Cell Cycle signaling* pathway. Earlier studies have reported abnormality in cell cycle phase in SLE patients [88]. Even though no pathway could be predicted in case of anti-ENA^+^ and anti-dsDNA^+^ENA^+^ SLE subsets due to small number of DEGs but they have unique expression pattern of genes as observed in this study for differentially expressed transcripts among distinct patient subsets.

In conclusion, this study has identified unique expression pattern of transcripts in SLE patients varying in autoimmune response to key nuclear autoantigens (dsDNA and ENA). We have also identified critical canonical pathways associated with dysregulated transcripts that may distinguish the patients with anti-ENA autoantibodies from the patients with autoantibodies against dsDNA. The possibility of underlying differences in the disease mechanism in SLE patients could be due to pathological role driven by distinct autoantibodies. The results of the present study in conjunction with the ongoing genomic analysis of SLE patients characterized on the basis of distinct end organ disease manifestations could generate useful information and provide avenues for development of new targeted and precise therapeutic interventions in SLE.

## Supporting Information

S1 Fig Correlation plots of differentially expressed transcripts among SLE patient samples within the group. A. Control samples B. Anti-dsDNA^+^ patient samples C. Anti-ENA^+^ patients and D. Anti-dsDNA^+^ENA^+^ patients.
(TIF)

S2 Fig Differential expressions of RNA species in distinct SLE subsets: x-axis depicts variation in the number of upregulated or down regulated transcripts belonging to five different classes of non-coding RNA like lincRNA, miRNA, snRNA, snoRNA and miscRNA in each subset of SLE patient. Error bars indicate the standard deviation.
(TIF)

S3 Fig Heat map representing the differentially expressed transcripts in each SLE patients as compared to controls. A. Anti-dsDNA^+^ patients B. Anti-ENA^+^ patients C. Anti-dsDNA^+^ENA^+^ patients. Rows in red shows the upregulation of transcripts and rows in green shows the downregulation of transcripts.
(TIF)

S4 Fig Graph representing variation of differentially expressed transcripts among individual samples and subgroups, as identified by RNA-seq analysis. A. CCL20 specifically upregulated in anti-dsDNA^+^ patients B. CXCL3 specifically upregulated in anti-dsDNA^+^ patients C. CCNA1 specifically upregulated in anti-ENA^+^ patients D. OPLAH specifically upregulated in anti-ENA^+^ patients E. EPHB2 specifically upregulated in anti-dsDNA^+^ENA^+^ patients F. IFNG specifically upregulated in anti-dsDNA^+^ENA^+^ patients.
(TIF)

S5 Fig Pattern recognition receptor in bacteria and viruses signaling pathway. The orange shaded molecules are the gene transcripts that are upregulated in anti-dsDNA^+^ SLE patients. The non-shaded nodes are the genes inferred by IPA from its knowledgebase.
(TIF)

S6 Fig Nur77 signaling in T lymphocytes pathway. The green shaded molecules are the gene transcripts that are downregulated in anti-dsDNA^+^ SLE patients. The non-shaded nodes are the genes inferred by IPA from its knowledgebase.
(TIF)

S7 Fig Complement signaling pathway. The orange shaded molecules are the gene transcripts that are upregulated in anti-ENA^+^ SLE patients. The non-shaded nodes are the genes inferred by IPA from its knowledgebase.
(TIF)

S8 Fig Actin cytoskeleton signaling pathway. The green shaded molecules are the gene transcripts that are downregulated in anti-ENA^+^ SLE patients. The non-shaded nodes are the genes inferred by IPA from its knowledgebase.
(TIF)

S9 Fig Antigen presentation pathway. The orange shaded molecules are the gene transcripts that are upregulated in anti-dsDNA^+^ENA^+^ SLE patients. The non-shaded nodes are the genes inferred by IPA from its knowledgebase.
(TIF)

S10 Fig Cyclin dependent kinases (CDK) 5 signaling pathway. The green shaded molecules are the gene transcripts that are downregulated in anti-dsDNA^+^ENA^+^ SLE patients. The non-shaded nodes are the genes inferred by IPA from its knowledgebase.
(TIF)

S11 Fig Interferon signaling pathway. The orange shaded molecules are the gene transcripts that are upregulated and the green shaded molecules are the gene transcripts that are downregulated in anti-ENA^+^ SLE patients. The non-shaded nodes are the genes inferred by IPA from its knowledgebase.
(TIF)

S12 Fig Cell cycle control of chromosomal replication pathway. The orange shaded molecules are the genes that are upregulated in anti-dsDNA^+^ SLE patients. The non-shaded nodes are the genes inferred by IPA from its knowledgebase.
(TIF)

S1 Table Read alignment summary of the RNA-sequencing data of SLE patient samples and control samples. (DOCX)

S2 Table Spread sheet representing differentially expressed transcripts in each SLE subsets belonging to various classes of RNA including coding RNA, non-coding RNA species, other transcripts (processed transcripts, pseudogene transcripts, antisense transcript etc.) and Ig gene transcripts. (XLSX)

S3 Table Functional analysis of uniquely expressed transcripts in distinct subsets of SLE patients A. Using GSEA tool B. Using DAVID bioinformatic database.
(DOCX)

S4 Table Distribution of various transcripts of interferon associated genes that are differentially expressed in distinct SLE subgroup. This excel spreadsheet contains a list of interferon associated transcripts along with ensemble ID and fold change.
(XLSX)

S5 Table Distribution of various transcripts of granulocyte associated genes that are differentially expressed in distinct SLE subgroup. This excel spreadsheet contains a list of granulocyte associated transcripts along with ensemble ID and fold change.
(XLSX)

S6 Table Spread sheet representing differentially expressed genes commonly identified by Cufflink and DESeq analysis program in each SLE patient subsets. (XLSX)

## References

[1] Yu C, Gershwin ME, Chang C. Diagnostic criteria for systemic lupus erythematosus: a critical review. J Autoimmun. 2014; 48–49: 10–3. 10.1016/j.jaut.2014.01.004 . 24461385

[2] Sherer Y, Gorstein A, Fritzler MJ, Shoenfeld Y. Autoantibody explosion in systemic lupus erythematosus: more than 100 different antibodies found in SLE patients. Semin Arthritis Rheum. 2004; 34(2):501–37. . 15505768 10.1016/j.semarthrit.2004.07.002

[3] Rus V, Atamas SP, Shustova V, Luzina IG, Selaru F, Magder LS, et al Expression of cytokine- and chemokine-related genes in peripheral blood mononuclear cells from lupus patients by cDNA array. Clin Immunol. 2002; 102(3):283–90. 10.1006/clim.2001.5182 11890715

[4] Han GM, Chen SL, Shen N, Ye S, Bao CD, Gu YY. Analysis of gene expression profiles in human systemic lupus erythematosus using oligonucleotide microarray. Genes Immun. 2003; 4(3):177–86. 10.1038/sj.gene.6363966 12700592

[5] Ye S, Pang H, Gu YY, Hua J, Chen XG, Bao CD, et al Protein interaction for an interferon-inducible systemic lupus associated gene, IFIT1. Rheumatology (Oxford). 2003; 42(10):1155–63. 10.1093/rheumatology/keg315 12777642

[6] Rus V, Chen H, Zernetkina V, Magder LS, Mathai S, Hochberg MC, et al Gene expression profiling in peripheral blood mononuclear cells from lupus patients with active and inactive disease. Clin Immunol. 2004; 112(3):231–4. 10.1016/j.clim.2004.06.005 15308115

[7] Nakou M, Knowlton N, Frank MB, Bertsias G, Osban J, Sandel CE, et al Gene expression in systemic lupus erythematosus: bone marrow analysis differentiates active from inactive disease and reveals apoptosis and granulopoiesis signatures. Arthritis Rheum. 2008; 58(11):3541–9. 10.1002/art.2396 . PMCID: PMC2760826. 18975309 PMC2760826

[8] Shi L, Zhang Z, Yu AM, Wang W, Wei Z, Akhter E, et al The SLE transcriptome exhibits evidence of chronic endotoxin exposure and has widespread dysregulation of non-coding and coding RNAs. PLoS One. 2014; 9(5):e93846 doi: 10.1371/journal.pone.0093846. eCollection 2014 . PMCID: PMC4010412. 24796678 10.1371/journal.pone.0093846 PMC4010412

[9] Zhao M, Liu S, Luo S, Wu H, Tang M, Cheng W, et al DNA methylation and mRNA and microRNA expression of SLE CD4+ T cells correlate with disease phenotype. J Autoimmun. 2014; 54:127–36. 10.1016/j.jaut.2014.07.002 . 25091625

[10] Rai G, Rai R, Saeidian AH, Rai M. Microarray to deep sequencing: Transcriptome and mirna profiling to elucidate molecular pathways in systemic lupus erythematosus. Immunol Res. 2015 10.1007/s12026-015-8672-y . 26188428

[11] Ellyard JI, Jerjen R, Martin JL, Lee AY, Field MA, Jiang SH, et al Identification of a pathogenic variant in TREX1 in early-onset cerebral systemic lupus erythematosus by Whole-exome sequencing. Arthritis Rheumatol. 2014; 66(12):3382–6. 10.1002/art.38824 25138095

[12] Van Eyck L, De Somer L, Pombal D, Bornschein S, Frans G, Humblet-Baron S, et al Brief Report: IFIH1 Mutation Causes Systemic Lupus Erythematosus With Selective IgA Deficiency. Arthritis Rheumatol. 2015; 67(6):1592–7. 10.1002/art.39110 . 25777993

[13] Chauhan SK, Singh VV, Rai R, Rai M, Rai G. Distinct autoantibody profiles in systemic lupus erythematosus patients are selectively associated with TLR7 and TLR9 upregulation. J Clin Immunol. 2013; 33(5):954–64. 10.1007/s10875-013-9887-0 . 23564191

[14] Rai R, Chauhan SK, Singh VV, Rai M, Rai G. Rai, Heat shock protein 27 and its regulatory molecule express differentially in SLE patients with distinct autoantibody profiles. Immunol Lett. 2015; 164(1):25–32. 10.1016/j.imlet.2015.01.007 . 25655337

[15] Chauhan SK, Singh VV, Rai R, Rai M, Rai G. Differential microRNA profile and post-transcriptional regulation exist in systemic lupus erythematosus patients with distinct autoantibody specificities. J Clin Immunol. 2014; 34(4):491–503. 10.1007/s10875-014-0008-5 24659206

[16] Chauhan SK, Rai R, Singh VV, Rai M, Rai G. Differential clearance mechanisms, neutrophil extracellular trap degradation and phagocytosis, are operative in systemic lupus erythematosus patients with distinct autoantibody specificities. Immunol Lett. 2015; 168(2):254–9. 10.1016/j.imlet.2015.09.016 . 26434792

[17] Gladman DD, Ibañez D, Urowitz MB. Systemic lupus erythematosus disease activity index 2000. J Rheumatol. 2002; 29(2):288–91. . 11838846

[18] Livak KJ, Schmittgen TD. Analysis of relative gene expression data using real-time quantitative PCR and the 2(-Delta Delta C(T)) Method. Methods. 2001; 25(4):402–8. 10.1006/meth.2001.1262 11846609

[19] Karapetyan AR, Buiting C, Kuiper RA, Coolen MW. Regulatory Roles for Long ncRNA and mRNA. Cancers (Basel). 2013; 5(2):462–90. 10.3390/cancers5020462 . PMCID: PMC3730338. 24216986 PMC3730338

[20] Jacobi AM, Reiter K, Mackay M, Aranow C, Hiepe F, Radbruch A, et al Activated memory B cell subsets correlate with disease activity in systemic lupus erythematosus: delineation by expression of CD27, IgD, and CD95. Arthritis Rheum. 2008; 58(6):1762–73. 10.1002/art.23498 . 18512812

[21] Jacobi AM, Odendahl M, Reiter K, Bruns A, Burmester GR, Radbruch A, et al Correlation between circulating CD27 high plasma cells and disease activity in patients with systemic lupus erythematosus. Arthritis Rheum. 2003; 48(5):1332–42. 10.1002/art.10949 . 12746906

[22] Tarte K, Zhan F, De Vos J, Klein B, Shaughnessy J Jr. Gene expression profiling of plasma cells and plasmablasts: toward a better understanding of the late stages of B-cell differentiation. Blood. 2003; 15;102(2):592–600. 10.1182/blood-2002-10-3161 . 12663452

[23] Lugar PL, Love C, Grammer AC, Dave SS, Lipsky PE. Molecular characterization of circulating plasma cells in patients with active systemic lupus erythematosus. PLoS One. 2012; 7(9):e44362 10.1371/journal.pone.0044362 . PMCID: PMC3448624. 23028528 PMC3448624

[24] Jacob N, Stohl W. Cytokine disturbances in systemic lupus erythematosus. Arthritis Res Ther. 2011; 13(4):228 10.1186/ar3349 . PMCID: PMC3239336. 21745419 PMC3239336

[25] Davas EM, Tsirogianni A, Kappou I, Karamitsos D, Economidou I, Dantis PC. Serum IL-6, TNFalpha, p55 srTNFalpha, p75srTNFalpha, srIL-2alpha levels and disease activity in systemic lupus erythematosus. Clin Rheumatol. 1999; 18(1):17–22. . 10088943 10.1007/s100670050045

[26] Gröndal G, Gunnarsson I, Rönnelid J, Rogberg S, Klareskog L, Lundberg I. Cytokine production, serum levels and disease activity in systemic lupus erythematosus. Clin Exp Rheumatol. 2000; 18(5):565–70. . 11072595

[27] Kidani Y, Bensinger SJ. Lipids rule: resetting lipid metabolism restores T cell function in systemic lupus erythematosus. J Clin Invest. 2014; 124(2):482–5. 10.1172/JCI74141 . PMCID: PMC3904634. 24463443 PMC3904634

[28] Moulton VR, Tsokos GC. Abnormalities of T cell signaling in systemic lupus erythematosus. Arthritis Res Ther. 2011; 13(2):207 10.1186/ar3251 . PMCID: PMC3132009. 21457530 PMC3132009

[29] Ivanov VN, Nikolić-Zugić J. Transcription factor activation during signal-induced apoptosis of immature CD4(+)CD8(+) thymocytes. A protective role of c-Fos. J Biol Chem. 1997; 272(13):8558–66. . 9079686

[30] Kyttaris VC, Zhang Z, Kampagianni O, Tsokos GC. Calcium signaling in systemic lupus erythematosus T cells: a treatment target. Arthritis Rheum. 2011; 63(7):2058–66. 10.1002/art.30353 . 21437870 PMC3128171

[31] Sipka S, Szucs K, Szántó S, Kovács I, Lakos G, Kiss E, et al Glucocorticosteroid dependent decrease in the activity of calcineurin in the peripheral blood mononuclear cells of patients with systemic lupus erythematosus. Ann Rheum Dis. 2001; 60(4):380–4. PMCID: PMC1753622. 10.1136/ard.60.4.380 11247869 PMC1753622

[32] Mosca M, Tani C, Carli L, Bombardieri S. Glucocorticoids in systemic lupus erythematosus. Clin Exp Rheumatol. 2011; 29(5 Suppl 68):S126–9. . 22018198

[33] Li Y, Zhao M, Yin H, Gao F, Wu X, Luo Y, et al Overexpression of the growth arrest and DNA damage-induced 45alpha gene contributes to autoimmunity by promoting DNA demethylation in lupus T cells. Arthritis Rheum. 2010; 62(5):1438–47. 10.1002/art.27363 . 20131288

[34] Maroder M, Bellavia D, Meco D, Napolitano M, Stigliano A, Alesse E, et al Expression of trKB neurotrophin receptor during T cell development. Role of brain derived neurotrophic factor in immature thymocyte survival. J Immunol. 1996; 157(7):2864–72. . 8816391

[35] Ikenouchi-Sugita A, Yoshimura R, Ueda N, Kodama Y, Umene-Nakano W, Nakamura J. Continuous decrease in serum brain-derived neurotrophic factor (BDNF) levels in a neuropsychiatric syndrome of systemic lupus erythematosus patient with organic brain changes. Neuropsychiatr Dis Treat. 2008; 4(6):1277–81. . PMCID: PMC2646658. 19337469 10.2147/ndt.s4259 PMC2646658

[36] Tamashiro LF, Oliveira RD, Oliveira R, Frota ER, Donadi EA, Del-Ben CM, et al Participation of the neutrophin brain-derived neurotrophic factor in neuropsychiatric systemic lupus erythematosus. Rheumatology (Oxford). 2014; 53(12):2182–90. 10.1093/rheumatology/keu251 . 24942492

[37] Kawano M, Seya T, Koni I, Mabuchi H. Elevated serum levels of soluble membrane cofactor protein (CD46, MCP) in patients with systemic lupus erythematosus (SLE). Clin Exp Immunol. 1999; 116(3):542–6. PMCID: PMC1905304. 10.1046/j.1365-2249.1999.00917.x 10361248 PMC1905304

[38] Alegretti AP, Schneider L, Piccoli AK, Monticielo OA, Lora PS, Brenol JC, et al Diminished expression of complement regulatory proteins on peripheral blood cells from systemic lupus erythematosus patients. Clin Dev Immunol. 2012; 2012:725684 10.1155/2012/725684 . PMCID: PMC3385850. 22761633 PMC3385850

[39] Bengtsson AA, Sturfelt G, Truedsson L, Blomberg J, Alm G, Vallin H, et al Activation of type I interferon system in systemic lupus erythematosus correlates with disease activity but not with antiretroviral antibodies. Lupus. 2000; 9(9):664–71. . 11199920 10.1191/096120300674499064

[40] Pascual V, Farkas L, Banchereau J. Systemic lupus erythematosus: all roads lead to type I interferons. Curr Opin Immunol. 2006; 18(6):676–82. 10.1016/j.coi.2006.09.014 17011763

[41] Zhang Z, Shi L, Song L, Ephrem E, Petri M, Sullivan KE. Interferon regulatory factor 1 marks activated genes and can induce target gene expression in systemic lupus erythematosus. Arthritis Rheumatol. 2015; 67(3):785–96. 10.1002/art.38964 . PMCID: PMC4342285. 25418955 PMC4342285

[42] Wu T, Qin X, Kurepa Z, Kumar KR, Liu K, Kanta H, et al Shared signaling networks active in B cells isolated from genetically distinct mouse models of lupus. J Clin Invest. 2007; 117(8):2186–96. PMCID: PMC1913486. 10.1172/JCI30398 17641780 PMC1913486

[43] Harley JB, James JA. Epstein-Barr virus infection induces lupus autoimmunity. Bull NYU Hosp Jt Dis. 2006; 64(1–2):45–50. . 17121489

[44] Poole BD, Scofield RH, Harley JB, James JA. Epstein-Barr virus and molecular mimicry in systemic lupus erythematosus. Autoimmunity. 2006; 39(1):63–70. 10.1080/08916930500484849 16455583

[45] Wu T, Xie C, Han J, Ye Y, Weiel J, Li Q, et al Metabolic disturbances associated with systemic lupus erythematosus. PLoS One. 2012; 7(6):e37210 10.1371/journal.pone.0037210 . PMCID: PMC3378560. 22723834 PMC3378560

[46] Tang Y, Ma X, Zhang H, Gu Z, Hou Y, Gilkeson GS, et al Gene expression profile reveals abnormalities of multiple signaling pathways in mesenchymal stem cell derived from patients with systemic lupus erythematosus. Clin Dev Immunol. 2012; 2012:826182 10.1155/2012/826182 . PMCID: PMC3433142. 22966240 PMC3433142

[47] Tsumiyama K, Takimoto M, Shiozawa S, Requirement of antigen cross-presentation for lupus tissue injuries: essential role of endosomal trafficking and proteosomal degradation. J Immunol. 2011; 186 (Meeting Abstract Supplement) 44.24.

[48] Jury EC, Flores-Borja F, Kalsi HS, Lazarus M, Isenberg DA, Mauri C, et al Abnormal CTLA-4 function in T cells from patients with systemic lupus erythematosus. Eur J Immunol. 2010; 40(2):569–78. 10.1002/eji.200939781 . 19950182

[49] Park YW, Kee SJ, Cho YN, Lee EH, Lee HY, Kim EM, et al Impaired differentiation and cytotoxicity of natural killer cells in systemic lupus erythematosus. Arthritis Rheum. 2009; 60(6):1753–63. 10.1002/art.24556 19479851

[50] Bezman NA, Cedars E, Steiner DF, Blelloch R, Hesslein DG, Lanier LL. Distinct requirements of micro RNAs in NK cell activation, survival, and function. J Immunol. 2010; 185(7):3835–46. 10.4049/jimmunol.1000980 . PMCID: PMC2943981. 20805417 PMC2943981

[51] Sullivan RP, Leong JW, Schneider SE, Keppel CR, Germino E, French AR, et al MicroRNA-deficient NK cells exhibit decreased survival but enhanced function. J Immunol. 2012; 188(7):3019–30. 10.4049/jimmunol.1102294 . PMCID: PMC3311726. 22379033 PMC3311726

[52] Seitz HM, Matsushima GK. Dendritic cells in systemic lupus erythematosus. Int Rev Immunol. 2010; 29(2):184–209. 10.3109/08830181003602507 . PMCID: PMC2892226. 20367140 PMC2892226

[53] Jeffries MA, Dozmorov M, Tang Y, Merrill JT, Wren JD, Sawalha AH. Genome-wide DNA methylation patterns in CD4+ T cells from patients with systemic lupus erythematosus. Epigenetics. 2011; 6(5):593–601. PMCID: PMC3121972. 10.4161/epi.6.5.15374 21436623 PMC3121972

[54] Perl A, Gergely P Jr, Banki K. Mitochondrial dysfunction in T cells of patients with systemic lupus erythematosus. Int Rev Immunol. 2004; 23(3–4):293–313. 10.1080/08830180490452576 15204090

[55] Perl A, Hanczko R, Doherty E. Assessment of mitochondrial dysfunction in lymphocytes of patients with systemic lupus erythematosus. Methods Mol Biol. 2012; 900:61–89. 10.1007/978-1-60761-720-4_4 . 22933065

[56] Akisaka T, Yoshida H, Inoue S, Shimizu K. Organization of cytoskeletal F-actin, G-actin, and gelsolin in the adhesion structures in cultured osteoclast. J Bone Miner Res. 2001; 16(7):1248–55. 10.1359/jbmr.2001.16.7.1248 11450700

[57] Frost PG, Lachmann PJ. The relationship of desoxyribonuclease inhibitor levels in human sera to the occurrence of antinuclear antibodies. Clin Exp Immunol. 1968; 3(5):447–55. . PMCID: PMC1578906. 5662583 PMC1578906

[58] Hadjiyannaki K, Lachmann PJ. The relation of deoxyribonuclease inhibitor levels to the occurrence of antinuclear antibodies in NZB-NZW mice. Clin Exp Immunol. 1972; 11(2):291–5. . PMCID: PMC1553626. 4557183 PMC1553626

[59] Hakkim A, Fürnrohr BG, Amann K, Laube B, Abed UA, Brinkmann V, et al Impairment of neutrophil extracellular trap degradation is associated with lupus nephritis. Proc Natl Acad Sci U S A. 2010; 107(21):9813–8. 10.1073/pnas.0909927107 . PMCID: PMC2906830. 20439745 PMC2906830

[60] Bennett L, Palucka AK, Arce E, Cantrell V, Borvak J, Banchereau J, et al Interferon and granulopoiesis signatures in systemic lupus erythematosus blood. J Exp Med. 2003; 197(6):711–23. PMCID: PMC2193846. 10.1084/jem.20021553 12642603 PMC2193846

[61] Banchereau R, Hong S, Cantarel B, Baldwin N, Baisch J, Edens M, et al Personalized Immunomonitoring Uncovers Molecular Networks that Stratify Lupus Patients. Cell. 2016; 165(3):551–65. 10.1016/j.cell.2016.03.008 27040498 PMC5426482

[62] Kirou KA, Lee C, George S, Louca K, Peterson MG, Crow MK. Activation of the interferon-alpha pathway identifies a subgroup of systemic lupus erythematosus patients with distinct serologic features and active disease. Arthritis Rheum. 2005; 52(5):1491–503. 10.1002/art.21031 15880830

[63] Niewold TB, Swedler WI. Systemic lupus erythematosus arising during interferon-alpha therapy for cryoglobulinemic vasculitis associated with hepatitis C. Clin Rheumatol. 2005; 24(2):178–81. 10.1007/s10067-004-1024-2 15565395

[64] Mohamed AAA, Md Yusof MY, El-Sherbiny Y, Emery P, Vital EM. Interferon Activity in Early and Established SLE: Interferon Score Is Lower in Early Disease and Not Seen without Antibodies to Extractable Nuclear Antigens [abstract]. Arthritis Rheumatol. 2015; 67 (suppl 10).

[65] Darrah E, Andrade F. NETs: the missing link between cell death and systemic autoimmune diseases? Front Immunol. 2012; 3:428 doi: 10.3389/fimmu.2012.00428. eCollection 2012 . PMCID: PMC3547286. 23335928 10.3389/fimmu.2012.00428 PMC3547286

[66] Leffler J, Martin M, Gullstrand B, Tydén H, Lood C, Truedsson L, et al Neutrophil extracellular traps that are not degraded in systemic lupus erythematosus activate complement exacerbating the disease. J Immunol. 2012; 188(7):3522–31. 10.4049/jimmunol.1102404 . 22345666

[67] Odendahl M, Jacobi A, Hansen A, Feist E, Hiepe F, Burmester GR, et al Disturbed peripheral B lymphocyte homeostasis in systemic lupus erythematosus. J Immunol. 2000; 165:5970–5979. . 11067960 10.4049/jimmunol.165.10.5970

[68] Huang W, Sinha J, Newman J, Reddy B, Budhai L, Furie R, et al The effect of anti-CD40 ligand antibody on B cells in human systemic lupus erythematosus. Arthritis Rheum. 2002; 46(6):1554–62. 10.1002/art.10273 . 12115186

[69] Subramanian S, Tus K, Li QZ, Wang A, Tian XH, Zhou J et al A Tlr7 translocation accelerates systemic autoimmunity in murine lupus. Proc Natl Acad Sci U S A. 2006; 27;103(26):9970–5. 10.1073/pnas.0603912103 . PMCID: PMC1502563. 16777955 PMC1502563

[70] Christensen SR, Kashgarian M, Alexopoulou L, Flavell RA, Akira S, Shlomchik MJ. Toll-like receptor 9 controls anti-DNA autoantibody production in murine lupus. J Exp Med. 2005; 18;202(2):321–31. 10.1084/jem.20050338 . PMCID: PMC2212997. 16027240 PMC2212997

[71] De La Torre M, Urra JM, Blanco J. Raised expression of cytokine receptor gp130 subunit on peripheral lymphocytes of patients with active lupus. A useful tool for monitoring the disease activity? Lupus. 2009; 18(3):216–22. 10.1177/0961203308096068 . 19213859

[72] Cai Z, Wong CK, Kam NW, Dong J, Jiao D, Chu M, et al Aberrant expression of regulatory cytokine IL-35 in patients with systemic lupus erythematosus. Lupus. 2015; 1257–66. 10.1177/0961203315585815 . 25966926

[73] Hrycek A, Kusmierz D, Dybała T, Swiatkowska L. Expression of messenger RNA for transforming growth factor-beta1 and for transforming growth factor-beta receptors in peripheral blood of systemic lupus erythematosus patients treated with low doses of quinagolide. Autoimmunity. 2007; 40(1):23–30. 10.1080/08916930601168093 17364494

[74] Beşliu AN, Pistol G, Marica CM, Bănică LM, Chiţonu C, Ionescu R, et al PI3K/Akt signaling in peripheral T lymphocytes from systemic lupus erythematosus patients. Roum Arch Microbiol Immunol. 2009; 68(2):69–79. . 20361524

[75] Kawasaki M, Fujishiro M, Yamaguchi A, Nozawa K, Kaneko H, Takasaki Y, et al Possible role of the JAK/STAT pathways in the regulation of T cell-interferon related genes in systemic lupus erythematosus. Lupus. 2011; 20(12):1231–9. 10.1177/0961203311409963 . 21980035

[76] Xiong W, Lahita RG. Novel treatments for systemic lupus erythematosus. Ther Adv Musculoskelet Dis. 2011; 3(5):255–66. 10.1177/1759720X11415456 . PMCID: PMC3383530. 22870484 PMC3383530

[77] Stylianou K, Petrakis I, Mavroeidi V, Stratakis S, Vardaki E, Perakis K, et al The PI3K/Akt/mTOR pathway is activated in murine lupus nephritis and downregulated by rapamycin. Nephrol Dial Transplant. 2011; 26(2):498–508. 10.1093/ndt/gfq496 . 20709738

[78] Cedeño S, Cifarelli DF, Blasini AM, Paris M, Placeres F, Alonso G, et al Defective activity of ERK-1 and ERK-2 mitogen-activated protein kinases in peripheral blood T lymphocytes from patients with systemic lupus erythematosus: potential role of altered coupling of Ras guanine nucleotide exchange factor hSos to adapter protein Grb2 in lupus T cells. Clin Immunol. 2003; 106(1):41–9. . 12584050 10.1016/s1521-6616(02)00052-9

[79] Becker AM, Dao KH, Han BK, Kornu R, Lakhanpal S, Mobley AB, et al SLE peripheral blood B cell, T cell and myeloid cell transcriptomes display unique profiles and each subset contributes to the interferon signature. PLoS One. 2013; 8(6):e67003 10.1371/journal.pone.0067003 . PMCID: PMC3691135. 23826184 PMC3691135

[80] Yin X, Knecht DA, Lynes MA. Metallothionein mediates leukocytechemotaxis. BMC Immunol. 2005; 6:21 PMCID: PMC1262721. 10.1186/1471-2172-6-21 16164753 PMC1262721

[81] Kakumanu P, Sobel ES, Narain S, Li Y, Akaogi J, Yamasaki Y, et al Citrulline dependence of anti-cyclic citrullinated peptide antibodies in systemic lupus erythematosus as a marker of deforming/erosive arthritis. J Rheumatol. 2009; 36(12):2682–90. 10.3899/jrheum.090338 . PMCID: PMC2803319. 19884269 PMC2803319

[82] Rupper A, Grove B, Cardelli J. Rab7 regulates phagosome maturation in dictyostelium. J Cell Sci. 2001; 114(Pt 13):2449–60. . 11559753 10.1242/jcs.114.13.2449

[83] Herrmann M, Voll RE, Zoller OM, Hagenhofer M, Ponner BB, Kalden JR. Impaired phagocytosis of apoptotic cell material by monocyte-derived macrophages from patients with systemic lupus erythematosus. Arthritis Rheum. 1998; 41(7):1241–50. 10.1002/1529-0131(199807)41:7<1241::AID-ART15>3.0.CO;2-H 9663482

[84] Ren Y, Tang J, Mok MY, Chan AW, Wu A, Lau CS. Increased apoptotic neutrophils and macrophages and impaired macrophage phagocytic clearance of apoptotic neutrophils in systemic lupus erythematosus. Arthritis Rheum. 2003; 48(10):2888–97. 10.1002/art.11237 14558095

[85] Huber AR, Kunkel SL, Todd RF 3rd, Weiss SJ. Regulation of transendothelial neutrophil migration by endogenous interleukin-8. Science. 1991; 254(5028):99–102. . 1718038 10.1126/science.1718038

[86] Seyednasrollah F, Laiho A, Elo LL. Comparison of software packages for detecting differential expression in RNA-seq studies. Brief Bioinform. 2015; 16(1):59–70. 10.1093/bib/bbt086 . PMCID: PMC4293378. 24300110 PMC4293378

[87] Zhang ZH, Jhaveri DJ, Marshall VM, Bauer DC, Edson J, Narayanan RK, et al A comparative study of techniques for differential expression analysis on RNA-Seq data. PLoS One. 2014; 13;9(8):e103207 doi: 10.1371/journal.pone.0103207. eCollection 2014 . PMCID: PMC4132098. 25119138 10.1371/journal.pone.0103207 PMC4132098

[88] Hara M, Kitani A, Harigai M, Hirose T, Norioka K, Hirose W, et al Differential abnormality in cell-cycle stage of peripheral B cells from patients with systemic lupus erythematosus. Rheumatol Int. 1987; 7(2):83–7. . 3497423 10.1007/BF00270312

