# Supplementary figures and images for: RNA-seq Analysis Reveals Unique Transcriptome Signatures in Systemic Lupus Erythematosus Patients with Distinct Autoantibody Specificities
Source: PLoS One. 2016 Nov 11;11(11):e0166312. doi: 10.1371/journal.pone.0166312 (PMC5106032; doi:10.1371/journal.pone.0166312)

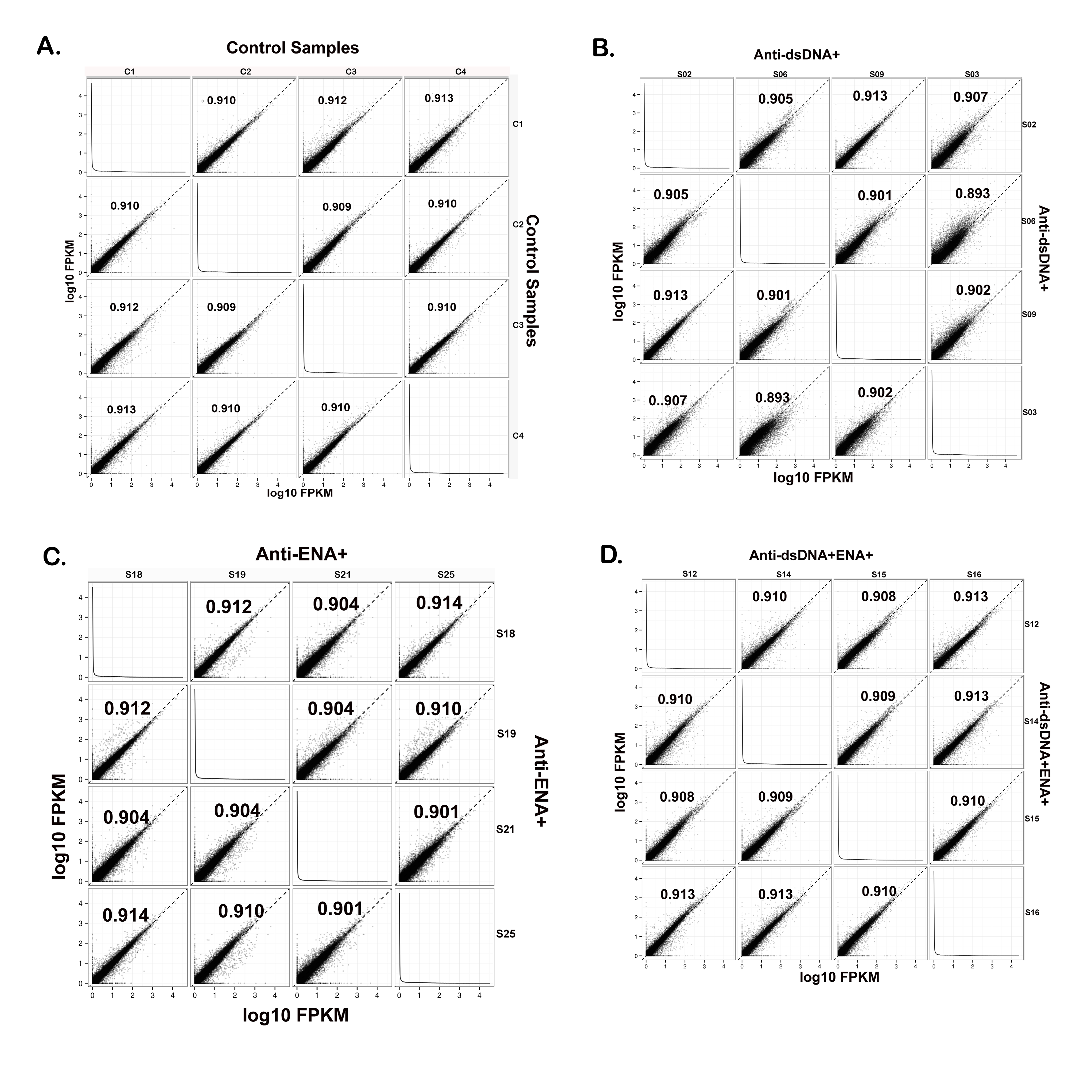

Supplement: S1 Fig — A. Control samples B. Anti-dsDNA+ patient samples C. Anti-ENA+ patients and D. Anti-dsDNA+ENA+ patients. (TIF) [file pone.0166312.s001.tif]

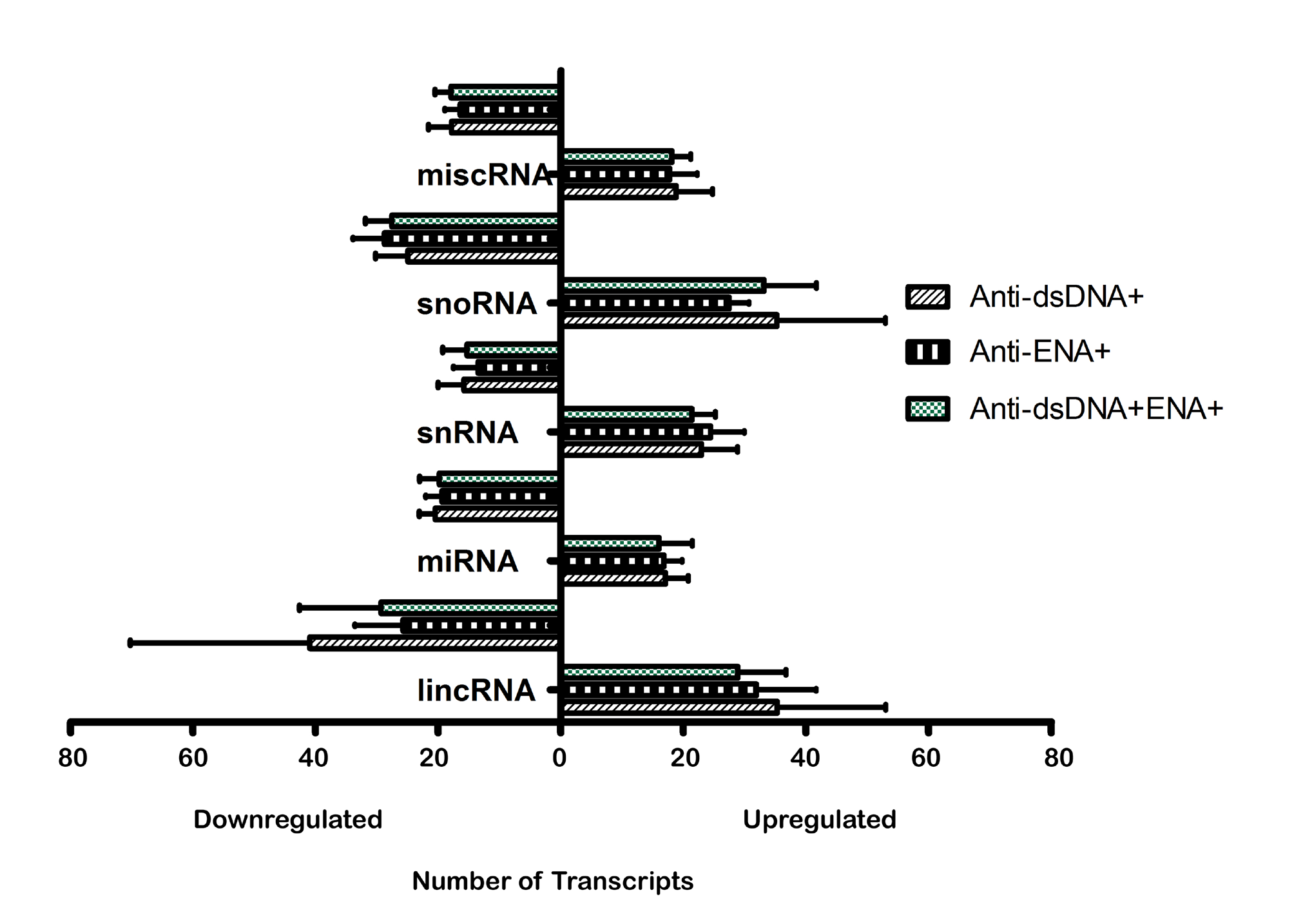

Supplement: S2 Fig — Error bars indicate the standard deviation. (TIF) [file pone.0166312.s002.tif]

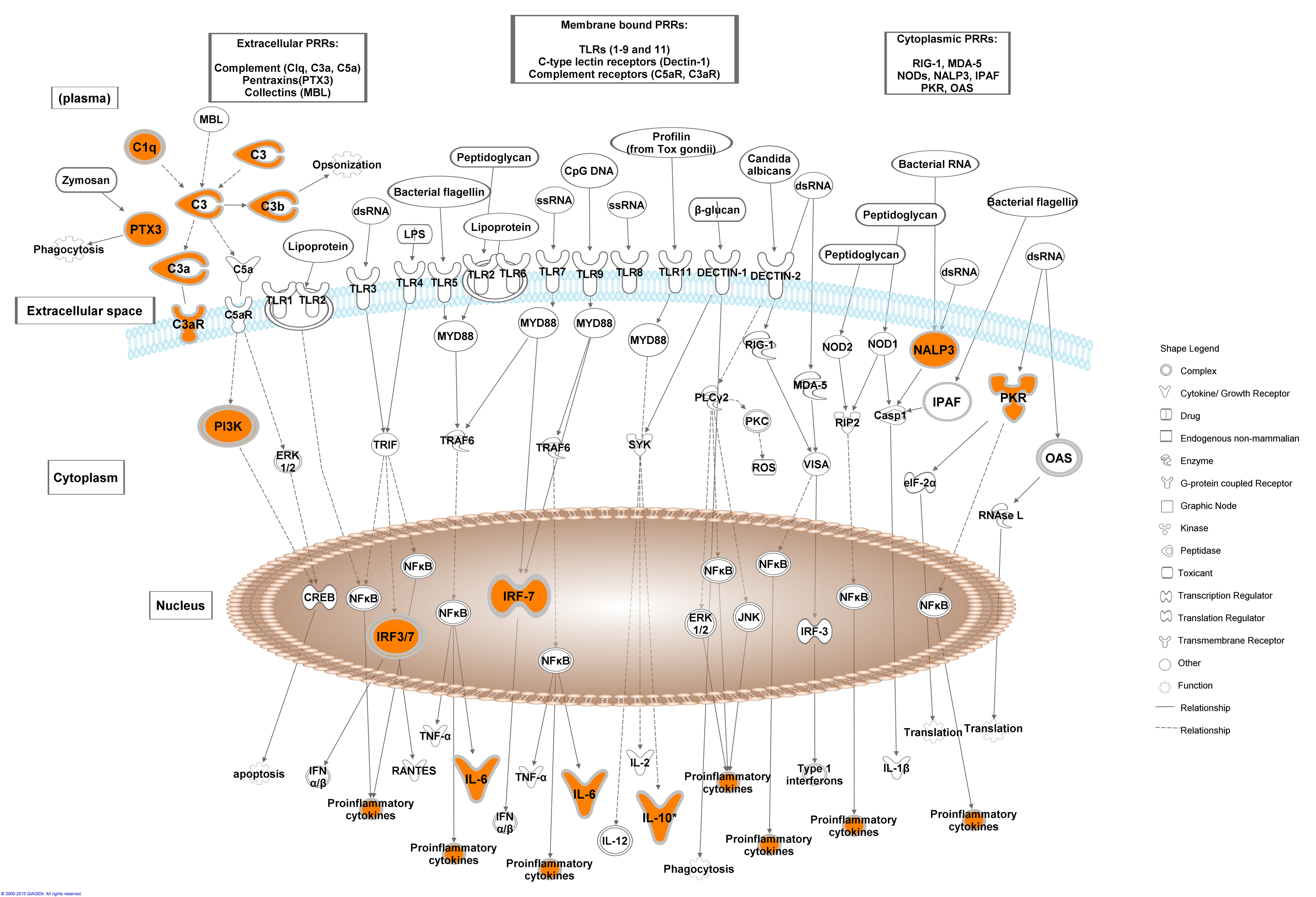

Supplement: S5 Fig — The orange shaded molecules are the gene transcripts that are upregulated in anti-dsDNA+ SLE patients. The non-shaded nodes are the genes inferred by IPA from its knowledgebase. (TIF) [file pone.0166312.s005.tif]

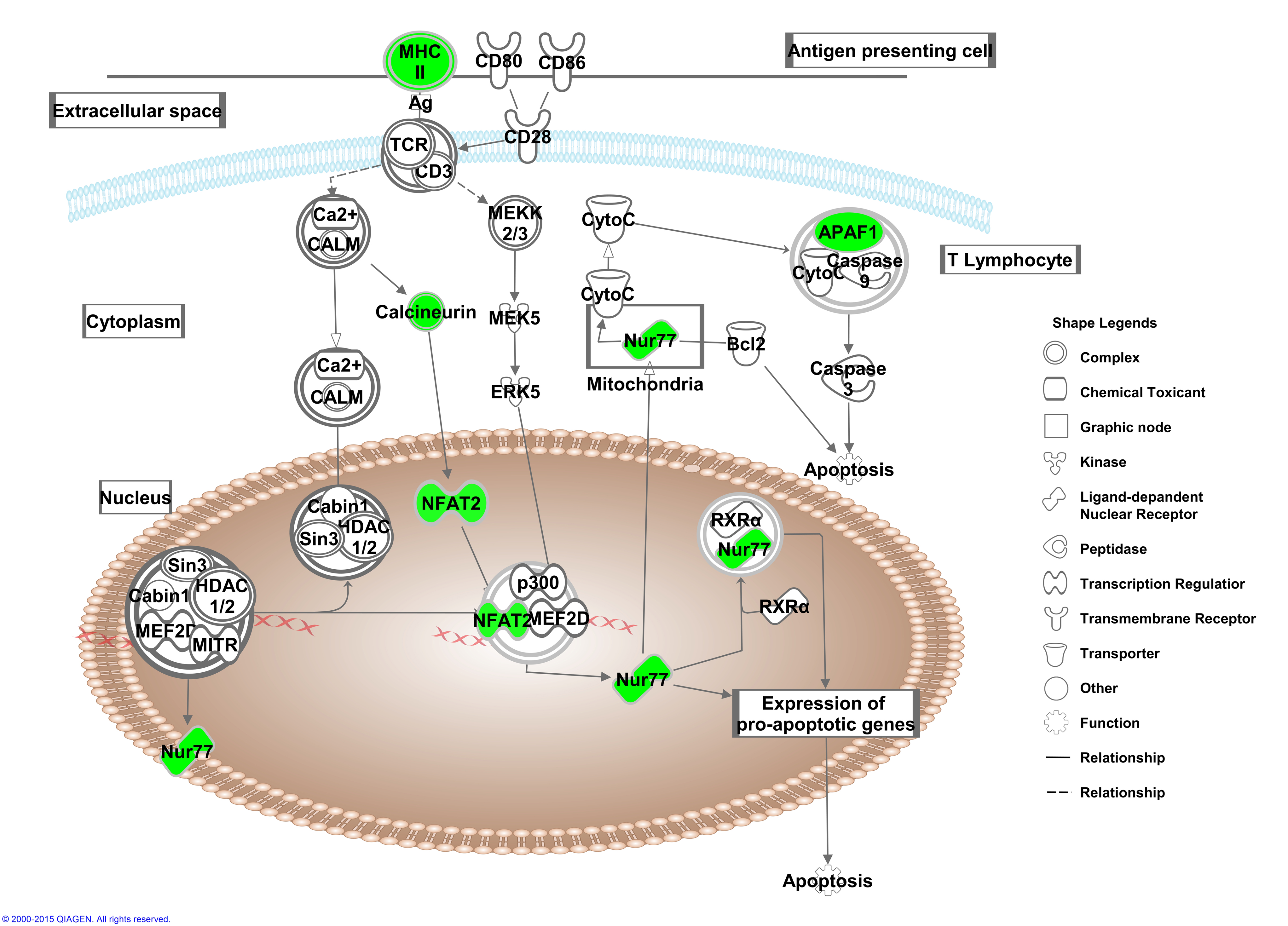

Supplement: S6 Fig — The green shaded molecules are the gene transcripts that are downregulated in anti-dsDNA+ SLE patients. The non-shaded nodes are the genes inferred by IPA from its knowledgebase. (TIF) [file pone.0166312.s006.tif]

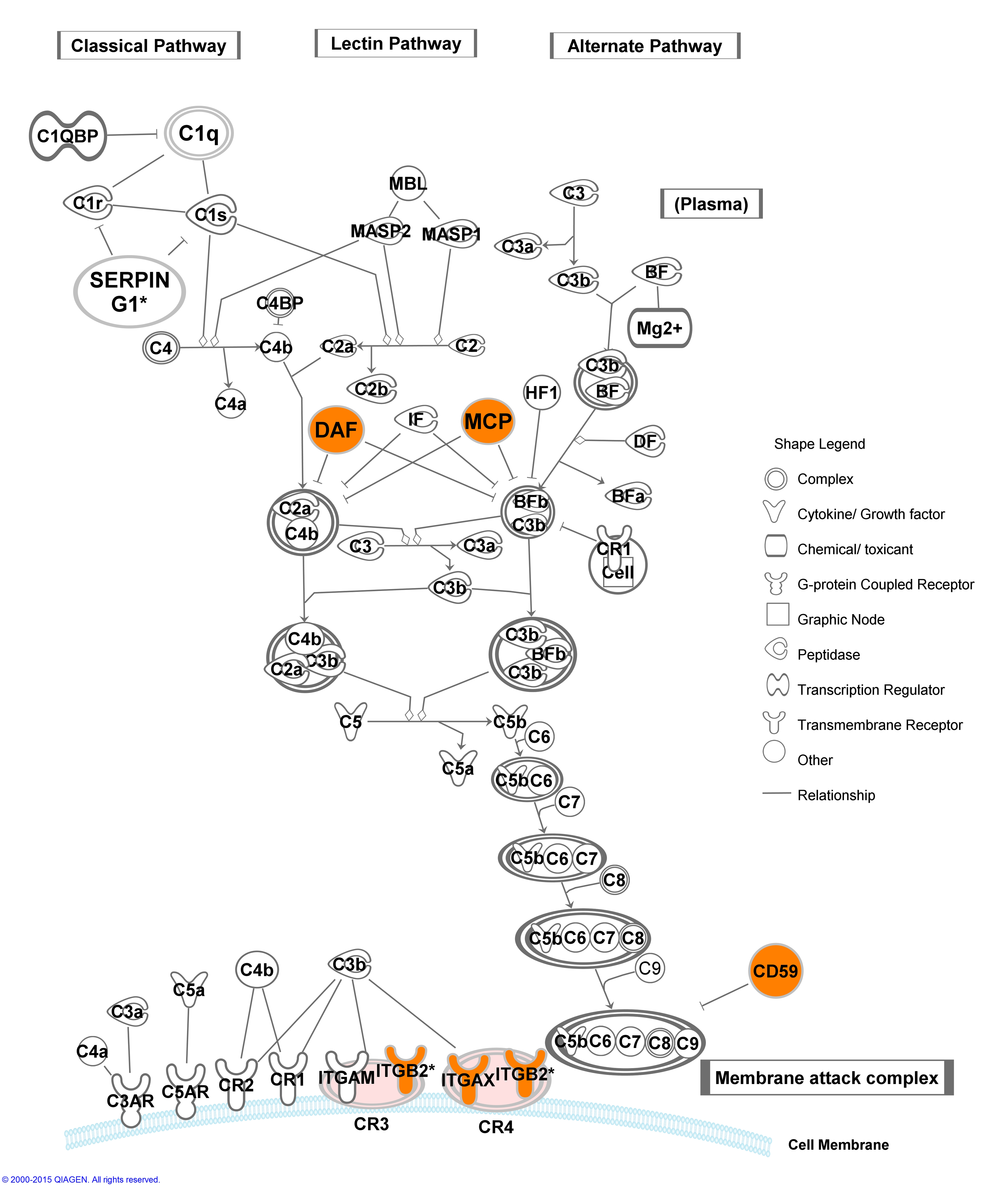

Supplement: S7 Fig — The orange shaded molecules are the gene transcripts that are upregulated in anti-ENA+ SLE patients. The non-shaded nodes are the genes inferred by IPA from its knowledgebase. (TIF) [file pone.0166312.s007.tif]

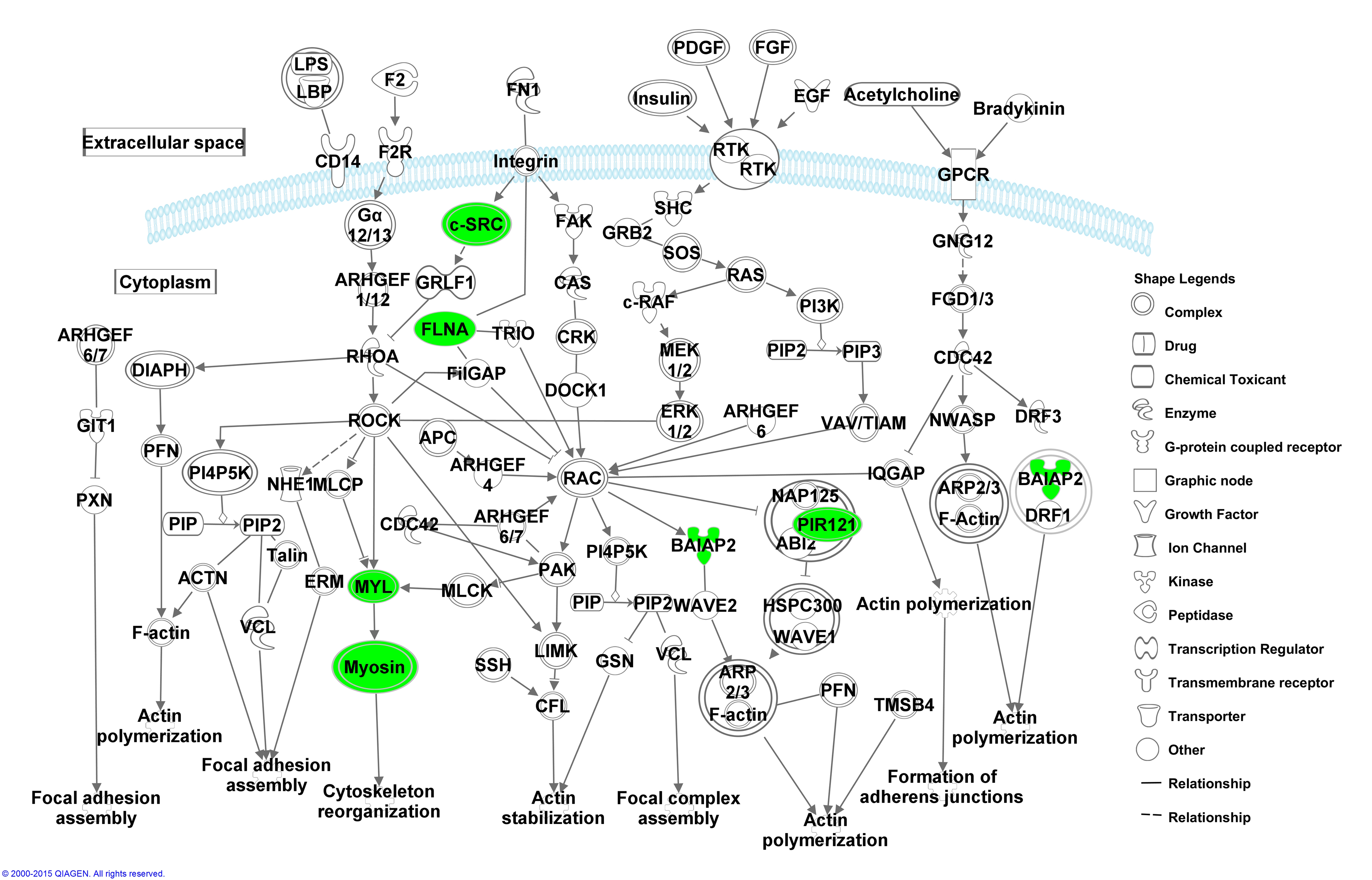

Supplement: S8 Fig — The green shaded molecules are the gene transcripts that are downregulated in anti-ENA+ SLE patients. The non-shaded nodes are the genes inferred by IPA from its knowledgebase. (TIF) [file pone.0166312.s008.tif]

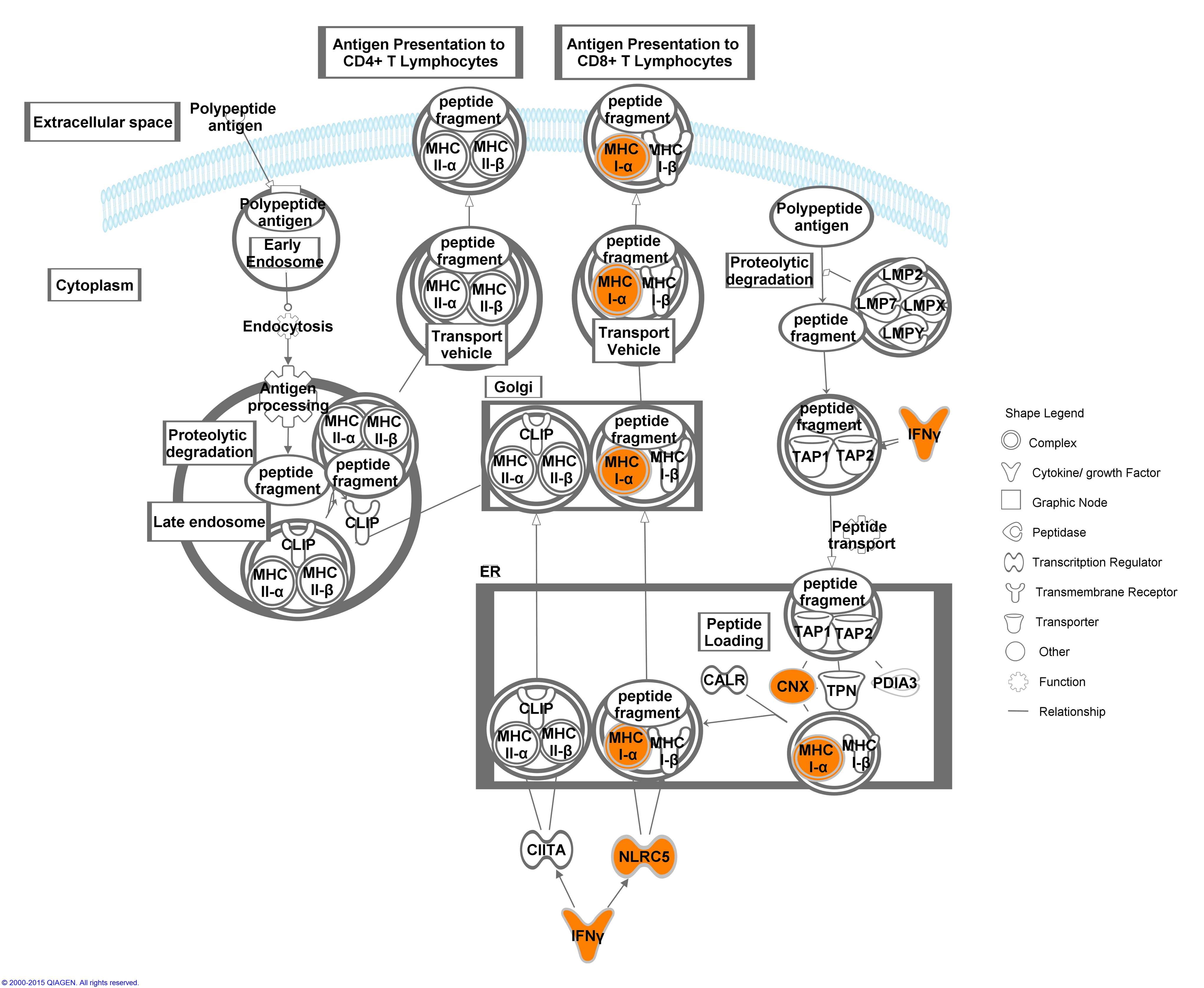

Supplement: S9 Fig — The orange shaded molecules are the gene transcripts that are upregulated in anti-dsDNA+ENA+ SLE patients. The non-shaded nodes are the genes inferred by IPA from its knowledgebase. (TIF) [file pone.0166312.s009.tif]

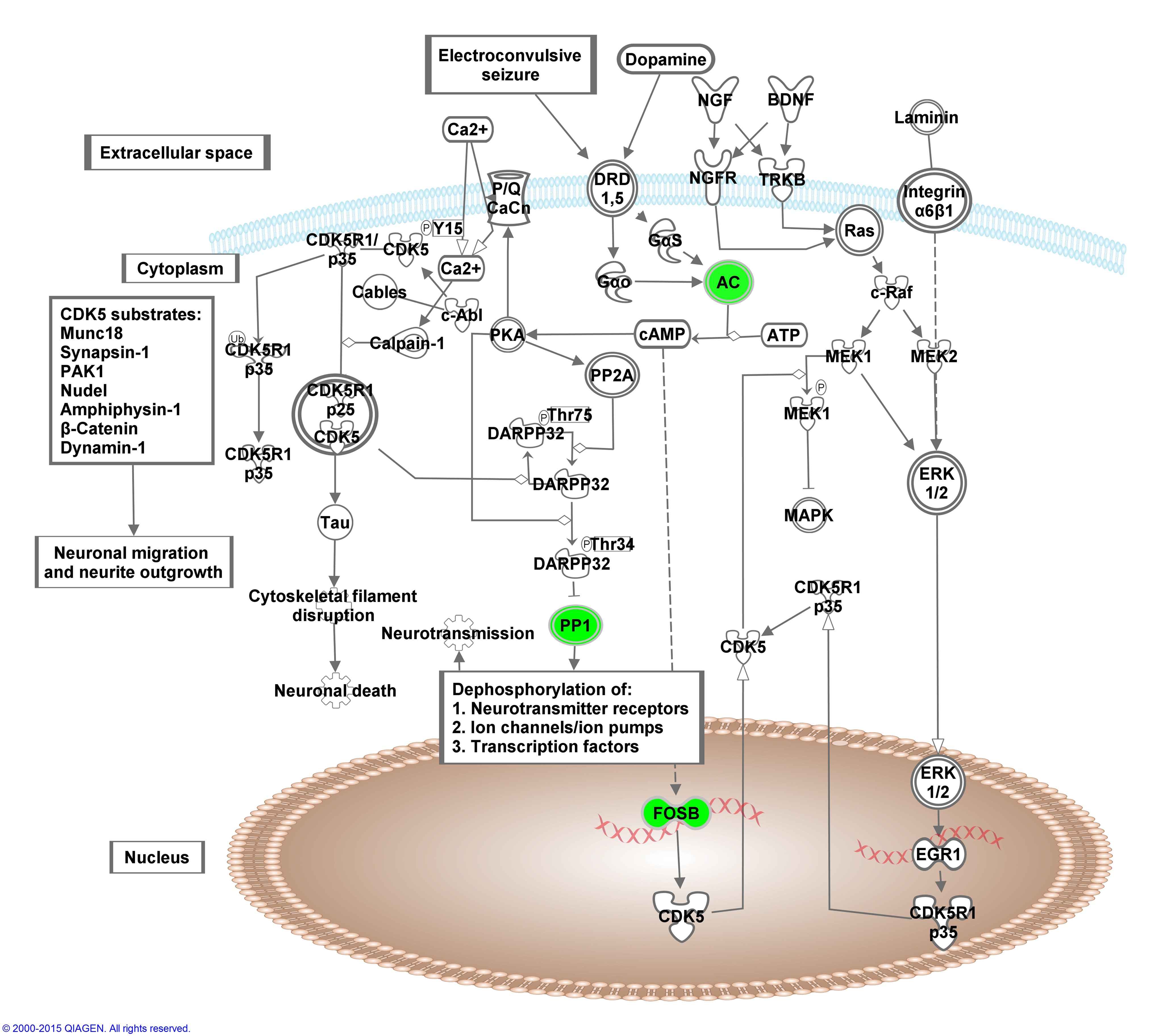

Supplement: S10 Fig — The green shaded molecules are the gene transcripts that are downregulated in anti-dsDNA+ENA+ SLE patients. The non-shaded nodes are the genes inferred by IPA from its knowledgebase. (TIF) [file pone.0166312.s010.tif]

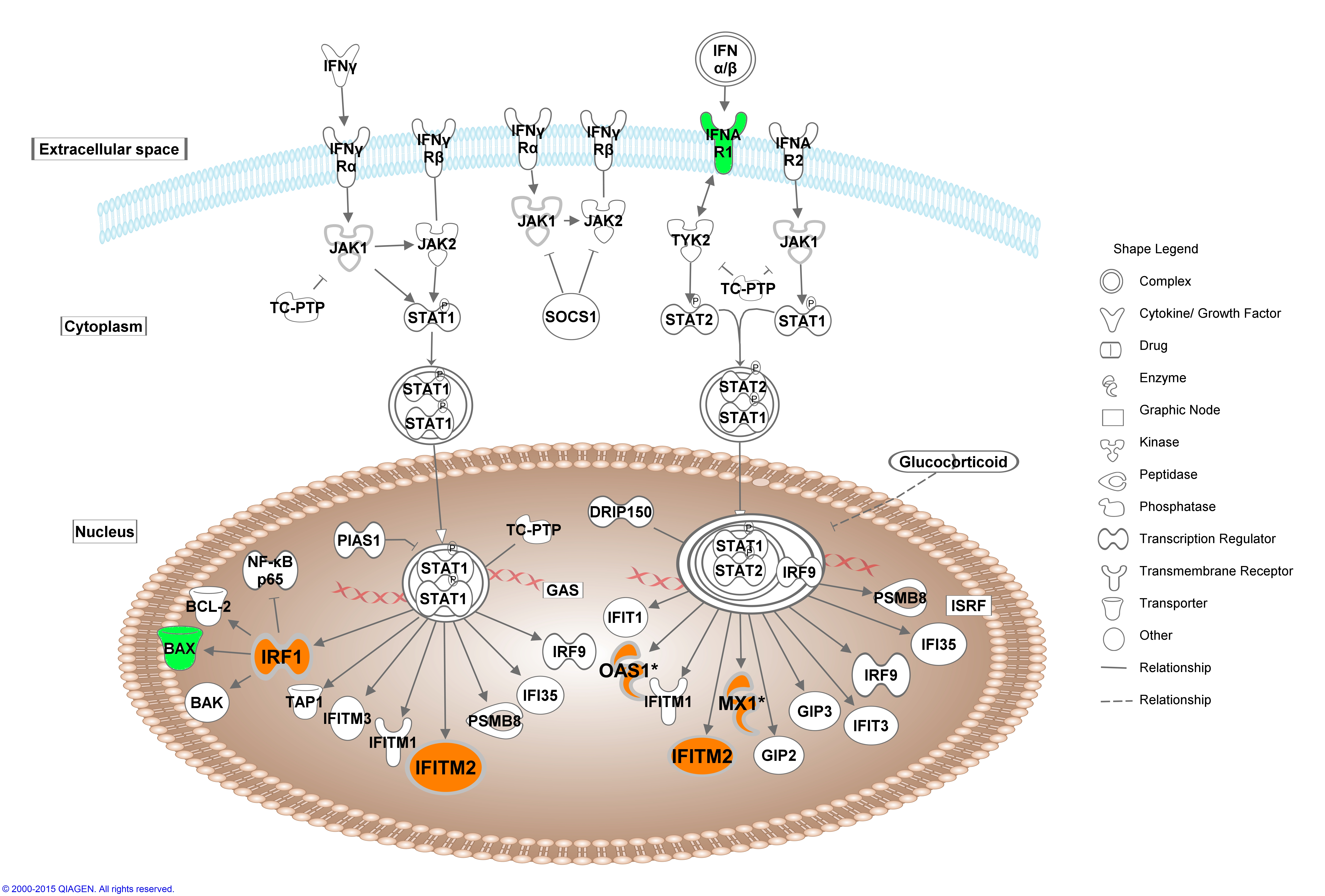

Supplement: S11 Fig — The orange shaded molecules are the gene transcripts that are upregulated and the green shaded molecules are the gene transcripts that are downregulated in anti-ENA+ SLE patients. The non-shaded nodes are the genes inferred by IPA from its knowledgebase. (TIF) [file pone.0166312.s011.tif]

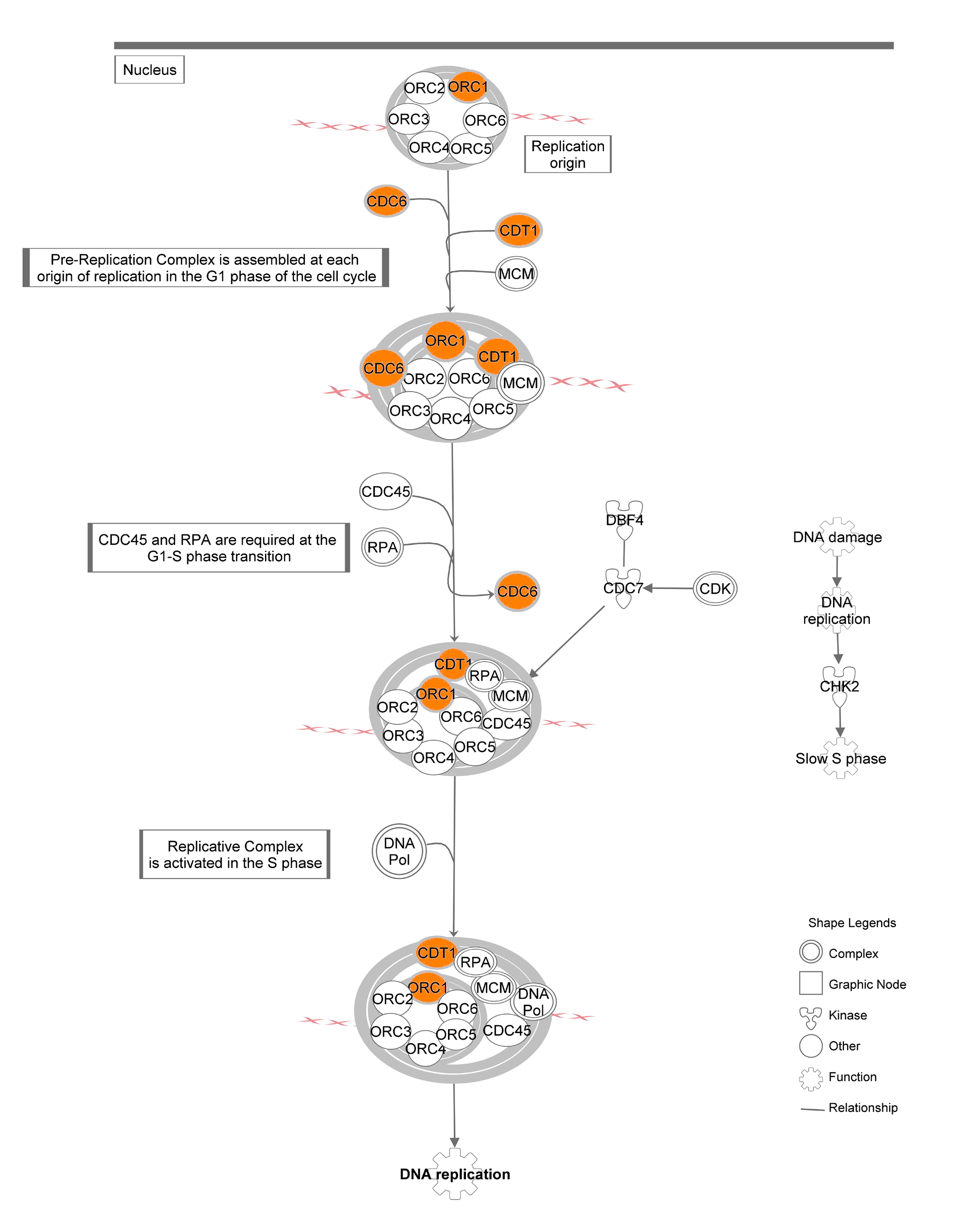

Supplement: S12 Fig — The orange shaded molecules are the genes that are upregulated in anti-dsDNA+ SLE patients. The non-shaded nodes are the genes inferred by IPA from its knowledgebase. (TIF) [file pone.0166312.s012.tif]
