# Supplementary material for: RNA-seq Analysis Reveals Unique Transcriptome Signatures in Systemic Lupus Erythematosus Patients with Distinct Autoantibody Specificities
Source: PLoS One. 2016 Nov 11;11(11):e0166312. doi: 10.1371/journal.pone.0166312 (PMC5106032; doi:10.1371/journal.pone.0166312)
Supplement: S1 Table — (DOCX) [file pone.0166312.s013.docx]

**S1 Table Read alignment summary**

| **Sample ID** | **Total**  **Reads** | **QC**  **Failed** | **QC**  **Failed**  **(%)** | **Aligned**  **Read**  **Count** | **Aligned**  **(%)** | **Unaligned**  **Read**  **Count** | **Unaligned (%)** |
| --- | --- | --- | --- | --- | --- | --- | --- |
| **S02** | 119,894,018 | 2,929,846 | 2.44% | 111,202,945 | 95.07% | 5,761,227 | 4.93% |
| **S03** | 75,128,218 | 2,535,012 | 3.37% | 64,576,341 | 88.96% | 8,016,865 | 11.04% |
| **S06** | 66,210,438 | 1,732,438 | 2.62% | 57,467,296 | 89.13% | 7,010,704 | 10.87% |
| **S09** | 89,644,180 | 17,616,542 | 19.65% | 64,272,930 | 89.23% | 7,754,708 | 10.77% |
| **S12** | 94,475,602 | 3,699,384 | 3.92% | 83,602,069 | 92.10% | 7,174,149 | 7.90% |
| **S14** | 79,948,040 | 3,087,856 | 3.86% | 69,382,697 | 90.27% | 7,477,487 | 9.73% |
| **S15** | 70,819,992 | 1,697,942 | 2.40% | 61,536,151 | 89.03% | 7,585,899 | 10.97% |
| **S16** | 96,730,640 | 2,886,306 | 2.98% | 84,645,371 | 90.20% | 9,198,963 | 9.80% |
| **S18** | 77,782,656 | 5,624,462 | 7.23% | 66,443,103 | 92.08% | 5,715,091 | 7.92% |
| **S19** | 71,443,006 | 3,066,752 | 4.29% | 60,934,059 | 89.12% | 7,442,195 | 10.88% |
| **S21** | 65,458,394 | 2,839,588 | 4.34% | 54,133,858 | 86.45% | 8,484,948 | 13.55% |
| **S25** | 69,708,560 | 3,665,592 | 5.26% | 59,441,227 | 90.00% | 6,601,741 | 10.00% |
| **C1** | 125,735,582 | 2,823,294 | 2.25% | 115,983,719 | 94.36% | 6,928,569 | 5.64% |
| **C2** | 61,247,558 | 2,592,442 | 4.23% | 48,869,206 | 83.32% | 9,785,910 | 16.68% |
| **C3** | 60,627,914 | 2,607,078 | 4.30% | 49,977,971 | 86.14% | 8,042,865 | 13.86% |
| **C4** | 61,754,480 | 2,805,548 | 4.54% | 51,865,974 | 87.98% | 7,082,958 | 12.02% |
