# Supplementary material for: RNA-seq Analysis Reveals Unique Transcriptome Signatures in Systemic Lupus Erythematosus Patients with Distinct Autoantibody Specificities
Source: PLoS One. 2016 Nov 11;11(11):e0166312. doi: 10.1371/journal.pone.0166312 (PMC5106032; doi:10.1371/journal.pone.0166312)
Supplement: S3 Table — Functional analysis of uniquely expressed transcripts in distinct subsets of SLE patients A. Using GSEA tool B. Using DAVID bioinformatic database. (DOCX) [file pone.0166312.s015.docx]

**S3 Table** **Functional analysis of uniquely expressed transcripts in distinct subsets of SLE patients**

1. **Gene Set Enrichment Analysis (GSEA)**

| **PATHWAY** | **P VALUE** | **RANK** |
| --- | --- | --- |
| **Anti-dsDNA^+^ SLE Patients** | | |
| TNFα Signaling via NFKB | 0.001 | 233 |
| G2M Checkpoint | 0.007 | 608 |
| DNA Repair | 0.033 | 472 |
| **Anti-ENA^+^ SLE Patients** | | |
| IFNα Response | 0.001 | 391 |
| IFNγ Response | 0.002 | 507 |
| Complement | 0.036 | 489 |
| **Anti-dsDNA^+^ENA^+^ SLE Patients** | | |
| Unfolded Protein Response | 0.038 | 48 |
| IFNγ Response | 0.092 | 402 |

1. **Database for Annotation, Visualization and Integrated Discovery (DAVID)**

| **GO TERM/ KEGG PATHWAY** | **P VALUE** | **FOLD ENRICHMENT** |
| --- | --- | --- |
| **Anti-dsDNA^+^ SLE Patients** | | |
| GO:0022403 Cell Cycle Phase | 1.98E-8 | 3.4 |
| GO:0006954 Inflammatory Response | 6.8E-7 | 3.5 |
| GO:0001817 Regulation of Cytokine Production | 7.5E-5 | 3.8 |
| KEGG Pathway: Cell Cycle | 0.002 | 3.6 |
| KEGG Pathway: NOD like Receptor Signaling | 0.032 | 4.06 |
| **Anti-ENA^+^ SLE Patients** |  |  |
| GO:0006220 Pyrimidine Nucleotide Metabolic process | 0.011 | 8.35 |
| GO:0002252 Immune Effector Process | 0.032 | 3.36 |
| GO:0016064 Immunoglobulin Mediated Immune Response | 0.034 | 5.56 |
| KEGG Pathway: p53 Signaling | 0.061 | 4.33 |
| **Anti-dsDNA^+^ENA^+^ SLE Patients** | | |
| GO:0019882 Antigen Processing and Presentation | 5.72E-05 | 21.76 |
| GO:0015629 Actin cytoskeleton | 0.018 | 3.32 |
| GO:0005739 Mitochondrion | 0.040 | 1.76 |
| KEGG Pathway: Antigen processing and presentation | 0.011 | 5.56 |
| KEGG Pathway: Natural killer cell mediated cytotoxicity | 0.012 | 4.17 |
